# Single spin exact gradients for the optimization of complex pulses and pulse sequences

**DOI:** 10.1007/s10858-025-00486-7

**Published:** 2026-02-17

**Authors:** Stella Slad, Burkhard Luy

**Affiliations:** 1 Institute for Biological Interfaces 4 – Magnetic Resonance, Karlsruhe Institute of Technology (KIT), Hermann-von-Helmholtz-Platz 1, Eggenstein-Leopoldshafen, 76344 Germany; 2 Institute of Organic Chemistry, Karlsruhe Institute of Technology (KIT), Fritz-Haber-Weg 6, Karlsruhe, 76131 Germany

**Keywords:** Shaped pulses, Optimal control, Exact gradient, Optimization, Analytical gradients, Holonomic constraints

## Abstract

The efficient computer optimization of magnetic resonance pulses and pulse sequences involves the calculation of a problem-adapted cost function as well as its gradients with respect to all controls applied. The gradients generally can be calculated as a finite difference approximation, as a GRAPE approximation, or as an exact function, e.g. by the use of the augmented matrix exponentiation, where the exact gradient should lead to best optimization convergence. The calculation of the exact gradient, on the other hand, is computationally demanding and is one of the determining factors for the overall time needed for optimization. Its improved calculation, e.g. by faster analytical solutions, is therefore highly desired. The majority of pulse optimizations involve a single spin 1/2, for which propagation is either represented by 3D-rotations or quaternions. Here, highly efficient analytical solutions for gradients for both cases are derived with respect to various possible controls. Controls are either *x* and *y* pulses, but also *z*-controls, as well as gradients with respect to amplitude and phase of a pulse shape. In addition, analytical solutions with respect to pseudo controls, involving holonomic constraints to maximum rf-amplitudes, maximum rf-power, or maximum rf-energy, are introduced. Using the hyperbolic tangent function, maximum values are imposed in a fully continuous and differentiable way. The obtained analytical gradients allow the calculation two orders of magnitude faster than the augmented matrix exponential approach, automatically speeding up any optimization in any optimization program by the same factor for the corresponding set of controls. The use of exact gradients for different controls is finally demonstrated in a number of optimizations involving broadband pulses of potential use in biomolecular applications for $^{15}$N, $^{13}$C, and $^{19}$F.

**Supplementary Information:**

The online version contains supplementary material available at 10.1007/s10858-025-00486-7.

## Introduction

Biomolecular NMR spectroscopy depends strongly on a large number of highly optimized shaped pulses, typically for selective or broadband excitation, inversion, and refocusing. Particularly ultra high field NMR spectroscopy poses requirements on shaped pulses that can only be fulfilled using the latest state-of-the-art optimization procedures, which themselves rely on efficient mathematical algorithms for convergence. A central and time-determining calculation in all modern optimization tools is the gradient of a given constant element of a shaped pulse with respect to the applicable controls, for example the *x*- and *y*-components of the pulse. Here, in this article, analytical solutions are introduced that can calculate such gradients for a single spin 1/2 and various types of controls with utmost efficiency and may thereby improve overall optimization times of applicable problems irrespective of a specific program used.

Due to spectroscopic requirements, computer optimization of pulses and pulse sequence building blocks has a long standing history in the NMR community. Starting out with composite pulses (Levitt and Freeman 1979; Levitt 1986; Lurie 1986; Tycko et al. 1985), shaped pulses (Emsley and Bodenhausen 1990, 1992; Ewing et al. 1990; Garwood and Ke 1991; Kupce and Freeman 1993, 1994; Smith et al. 2001; Tannús and Garwood 1997; Zax et al. 1988), heteronuclear decoupling (Levitt et al. 1982; Shaka et al. 1983) and Hartmann-Hahn-transfer building blocks (Kadkhodaie et al. 1991; Shaka et al. 1988), the success of todays liquid state NMR spectroscopy is largely built on these elements. With the advent of optimal control based algorithms (Conolly et al. 1986; Mao et al. 1986; Rosenfeld and Zur 1996) and in particular with the GRAPE algorithm (de Fouquieres et al. 2011; Khaneja et al. 2005; Skinner et al. 2003), possibilities in pulse design increased dramatically in the past two decades and examples in pulse shape design (Ehni and Luy 2013; Ehni et al. 2022; Ehni and Luy 2014, 2012; Gershenzon et al. 2008, 2007; Haller et al. 2022; Kobzar et al. 2005; Koos et al. 2015, 2017; Martikyan et al. 2021; Slad et al. 2022; Spindler et al. 2012, 2017; Odedra and Wimperis 2012; Spindler et al. 2013; Joseph and Griesinger 2023; Buchanan et al. 2025; He et al. 2024) show utmost performance close to the physical limits (Kobzar et al. 2004, 2008, 2012; Lapert et al. 2012). However, even larger bandwidths and/or lower rf-energies will have to be explored as well as extremely complex optimizations like whole heternuclear decoupling periods (Neves et al. 2009; Schilling et al. 2014), that easily lead to very long optimization times lasting weeks to months on highly parallelized supercomputers. It is therefore worth looking into the basic mathematics to look for analytical solutions of spin system treatment wherever possible to significantly speed up corresponding calculations.

The majority of current pulse and pulse sequence optimizations are performed using a single spin in Liouville superoperator (Hogben et al. 2011) or Bloch space (Bonnard et al. 2012; Skinner et al. 2004). This description is perfectly suited for optimizing the transfer from an intial to a final state, as e.g. the case for so-called point-to-point pulses (Kobzar et al. 2004; Skinner et al. 2003; Kobzar et al. 2008). Other optimizations, like having a defined propagator in universal rotation pulses (Khaneja et al. 2005; Kobzar et al. 2012) as a target, are better described using either Cayley-Klein parameters (Cayley 1997) or the related quaternion formalism (Hamilton 1843; Rodrigues 1840). In each type of optimization, a Hamiltonian needs to be diagonalized, which can be done by numerical exponentiation using for example the Padé algorithm, or by analytical formulae, which are particularly straightforward in the case of a single spin in Bloch space, where this is resembled by well-studied three dimensional Cartesian rotation matrices. In addition, partial derivatives to all controls need to be calculated in gradient and hessian based optimization algorithms. This can be achieved by a linear approximation as in the original GRAPE formalism (Khaneja et al. 2005; Skinner et al. 2003), but exact gradients are more efficient for the convergence of optimizations (de Fouquieres et al. 2011; Kuprov 2023). Such exact gradients can be calculated using a general approach described by Aizu (1963); Levante et al. (1996), or by a recent development in algebra based on the matrix exponentiation of co-propagators (Goodwin and Kuprov 2015, 2016). For a single spin, however, analytical solutions with respect to conventional controls were recently introduced with the ESCALADE approach (Foroozandeh and Singh 2021) and it was shown that very fast optimizations are possible based on a mixture of exact analytical gradients and numerical hessian calculations (Goodwin and Vinding 2023).

The goal of computational optimization in NMR spectroscopy must be to be able to address more and more complex problems. In this publication we focus on the improvement of extremely complex optimizations that can be performed with single spin calculations. One goal is, for example, to achieve broadband pulses that can cover bandwidths 20 to 100 times the applied maximum rf-amplitude to address the full chemical shift bandwidths of nuclei relevant for biological and pharmaceutical problems like $^{15}$N, $^{19}$F, $^{31}$P, as well as other nuclei of broader chemical interest like $^{119}$Sn, or $^{195}$Pt. In such cases, computational demands rise non-linearly and while short pulse shapes can be optimized in a matter of seconds to minutes on a conventional PC, the desired demanding pulses may take months on high level supercomputers or are not at all feasible with current technology due to limitations in memory. This memory issue is particularly pressing in the case of hessian-based optimization algorithms. If, for example, a 10 ms pulse with 0.1 $\mu$s digitization is considered, 100.000 digits result in 200.000 controls (x and y component), which in turn lead to a 200.000$\times$200.000 hessian matrix with double precision numbers, which corresponds to 320 GB memory for the calculation of a single condition hessian in an optimization. As our aim is at this type of optimization complexity, we do not consider second derivative hessian matrices in the following and reduce our view to exact gradients only, which can be calculated much more memory efficient and are readily combined with e.g. the Limited-memory Broyden–Fletcher–Goldfarb–Shanno (LBFGS) optimization algorithm. In this context, we here derive analytical derivatives in the Bloch picture based on rotational matrices, as well as for four-dimensional quaternions for propagator-based optimizations in a tabular form. It is thereby closely related to the ESCALADE approach, for which a very nice abstract way of gradient and hessian calculations based on the Rodrigues expansion has been developed (Foroozandeh and Singh 2021). In the present approach, however, we use a much simpler method in which the quaternions and the Rodrigues formula for rotations are directly used to derive gradients simply from individual matrix components. We therefore can extent the ESCALADE approach to more individualized optimization concepts: next to the conventional case with *x* and *y*-controls, we also consider several special cases, like the formulation in Cartesian coordinates for *x*,*y* pulses including *z*-controls, or the equivalent description using polar amplitude, phase and *z*-controls for pulse shapes. Also direct derivatives for holonomic constraints using $\tanh$ based pseudo-parameters for amplitude, power, and energy-limited pulse optimizations are derived.

## Theory

### GRAPE algorithm formulation for a single spin 1/2

#### Optimization of point-to-point pulses

A shaped pulse can be seen as a sequence of *N* short pulses of length $\Delta t$ with piecewise constant rf-amplitudes, where the $j^{\textrm{th}}$ pulse normally consists of two controls $\omega _{\textrm{x}}(j)$ and $\omega _{\textrm{y}}(j)$ that represent the *x* and *y*-components of the shaped pulse. While the offset frequency $\omega _{\textrm{off}}$ at a certain position in the spectrum relative to the irradiation frequency constitutes the free evolution or drift Hamiltonian

1 $$\begin{aligned} {\mathcal {H}}_0 = \omega _{\textrm{off}} \ I_z = 2 \pi \ \nu _{\textrm{off}} \ I_z, \end{aligned}$$

the pulses constitute the control Hamiltonian at the $j^{\textrm{th}}$ pulse

2 $$\begin{aligned}& {\mathcal {H}}_{\textrm{1}}(j) = \omega _{\textrm{x}}(j) \ I_x + \omega _{\textrm{y}}(j) \ I_y \\ & \quad \qquad= 2 \pi \ \nu _{\textrm{rf}}(j) \{ \cos \alpha (j) \ I_x + \sin \alpha (j) \ I_y \}. \end{aligned}$$

For a given sequence of *N* pulses with duration $\Delta t$ at a specific offset $\nu _{\textrm{off}}$ the propagator is given by

3 $$\begin{aligned} U_j = \exp \{ - i \{ {\mathcal {H}}_0 + {\mathcal {H}}_{\textrm{1}}(j) \} \Delta t \} \end{aligned}$$

and the propagation of an initial spin density operator $\rho _0$ can be written as

4 $$\begin{aligned} \rho _N = U_N \cdots U_j \cdots U_1 \ \rho _0 \ U^{\dagger }_1 \cdots U^{\dagger }_j \cdots U^{\dagger }_N . \end{aligned}$$

The goal of a point-to-point optimization is to find values of the controls that minimize the differences between the desired final state of the spin ($\mathrm {\lambda }_{\textrm{F}}$) and the obtained final density operator with the current control amplitudes ($\mathrm {\rho }_N$), which is identical to maximizing their overlap according to (Khaneja et al. 2005)

5 $$\begin{aligned} \Phi _{\textrm{PP}}= Re \left\langle {\lambda _{\textrm{F}}}\left| \right. {\rho _{{N}}}\right\rangle \end{aligned}$$

In order to maximize the cost function $\Phi _{\textrm{PP}}$, we have to minimize its gradient with respect to every control at every timestep, which for the $j^{th}$ time point is resulting in

6 $$\begin{aligned} & \Gamma _{\textrm{PP}}(j)=\begin{pmatrix}\dfrac{\partial \Phi _{\textrm{PP}}}{\partial \omega _x(j)} \\ \dfrac{\partial \Phi _{\textrm{PP}}}{\partial \omega _y(j)} \end{pmatrix} \nonumber \\ & \quad = \begin{pmatrix} \left\langle {\lambda _j}\bigg |{ \dfrac{\partial U_j}{\partial \omega _x(j)} \rho _{j-1} U^{\dagger }_j}\right\rangle + \left\langle {\lambda _j}\bigg |{ U_j \rho _{j-1} \dfrac{\partial U^{\dagger }_j}{\partial \omega _x(j)}}\right\rangle \\ \left\langle {\lambda _j}\bigg |{ \dfrac{\partial U_j}{\partial \omega _y(j)} \rho _{j-1} U^{\dagger }_j}\right\rangle + \left\langle {\lambda _j}\bigg |{ U_j \rho _{j-1} \dfrac{\partial U^{\dagger }_j}{\partial \omega _y(j)}}\right\rangle \end{pmatrix}\nonumber \\ & \quad \approx \begin{pmatrix}\left\langle {\lambda _j}\left| \right. { - i \Delta t [{\mathcal {H}}_{1_x}(j), \rho _j]}\right\rangle \\ \left\langle {\lambda _j}\left| \right. { - i \Delta t [{\mathcal {H}}_{1_y}(j), \rho _j]}\right\rangle \end{pmatrix} \end{aligned}$$

with $\lambda _j = U^{\dagger }_{j+1} \cdots U^{\dagger }_{N} \ \lambda _{\textrm{F}} U_N \cdots U_{j+1}$, $\rho _j = U_j \cdots U_1 \ \rho _0 \ U^{\dagger }_1 \cdots U^{\dagger }_j$ and ${\mathcal {H}}_{1_x}(j)$ and ${\mathcal {H}}_{1_y}(j)$ being the *x* and *y* components of the control Hamiltonian. Thus, for each iteration *i* of the optimization, the controls $\omega ^{\textrm{}}_{{k}}(j)$, $k\in \{x,y\}$, are guaranteed to increase the cost function $\Phi _{\textrm{PP}}$ for an infinitesimal $\epsilon$ according to

7 $$\begin{aligned} \omega _{{k}}^{(i+1)}(j) \rightarrow \omega _{{k}}^{(i)}(j)+\epsilon \frac{\partial \Phi _{\textrm{PP}}}{\partial \omega _{{k}}(j)}, \end{aligned}$$

until convergence to a (local) optimum is reached. Please note that the partial derivatives may also be calculated using the rotation angles

8 $$\begin{aligned} \theta _x(j) = \omega _x(j) \, \Delta t \, \, ; \, \, \theta _y(j) = \omega _y(j) \, \Delta t. \end{aligned}$$

In this case, the update can be rewritten into

9 $$\begin{aligned} \theta _{{k}}^{(i+1)}(j) \rightarrow \theta _{{k}}^{(i)}(j)+\epsilon \frac{\partial \Phi _{\textrm{PP}}}{\partial \theta _{{k}}(j)}, \end{aligned}$$

using the relation $d\omega / d\theta = 1/\Delta t$. Instead of the GRAPE-approximation given at the end of Eq. 6, it is advantageous to use the computationally more costly exact gradients for the pulse sequence update (de Fouquieres et al. 2011). The critical point in these exact gradients is the calculation of $\partial U_j / \partial \omega _x(j)$ or $\partial U_j / \partial \theta _x(j)$, respectively, and its complex conjugate, for which several solutions have been proposed (Goodwin and Kuprov 2015; Levante et al. 1996), but elegant analytical solutions might be of particular interest for specific problems. For a single spin we will derive such very compact analytical forms for various optimization types in the following.

In order to design broadband PP pulses with $B_1$ inhomogeneity compensation, it is further necessary to calculate the cost function and the gradient for $n_{\textrm{off}}$ offsets linearly distributed over the desired bandwdith $\Delta \nu$ and $n_{\textrm{rf}}$ different rf-amplitudes $\nu _{\textrm{rf}}$ covering the desired $B_1$-compensated range. The global quality factor and the global gradient can be calculated as averages according to

10 $$\begin{aligned} \overline{\Phi _{\textrm{PP}}}=\frac{1}{n_{\textrm{off}}n_{\textrm{rf}}}\displaystyle \sum _{i=1}^{n_{\textrm{off}}}\sum _{l=1}^{n_{\textrm{rf}}} \Phi _{\textrm{PP}}(\nu ^i_{\textrm{off}},\nu ^l_{\textrm{rf}}) \end{aligned}$$

and

11 $$\begin{aligned} \overline{\Gamma _{\textrm{PP}}(j)}=\frac{1}{n_{\textrm{off}}n_{\textrm{rf}}}\sum _{i=1}^{n_{\textrm{off}}}\sum _{l=1}^{n_{\textrm{rf}}} \Gamma _{\textrm{PP}}(j,\nu ^i_{\textrm{off}},\nu ^l_{\textrm{rf}}) . \end{aligned}$$

#### Optimization of universal rotation pulses

If not the transformation of a given state to a final state, but rather the rotation in Hilbert space itself is the target, the propagator itself can be optimized (de Fouquieres et al. 2011; Khaneja et al. 2005; Kobzar et al. 2012; Luy et al. 2005; Skinner et al. 2012). Considering the total propagator *U*(*T*) at time point $T = N \Delta t$ given by

12 $$\begin{aligned} U(T) = U_N \cdots U_j \cdots U_1, \end{aligned}$$

a cost function with respect to the desired propagator $U_{\textrm{F}}$ can be formulated as

13 $$\begin{aligned} \Phi _{\textrm{UR}} = Re\left\langle {U_{\textrm{F}}}\left| \right. {U(T)}\right\rangle , \end{aligned}$$

and the corresponding gradients to the $j^{th}$ time step for *x* and *y* components of the pulse train s are approximated by (Khaneja et al. 2005)

14 $$\begin{aligned} & \Gamma _{\textrm{UR}}(j)=\begin{pmatrix}\dfrac{\partial \Phi _{\textrm{UR}}}{\partial \theta _x(j)} \\ \dfrac{\partial \Phi _{\textrm{UR}}}{\partial \theta _y(j)} \end{pmatrix} = \begin{pmatrix}Re \left\langle {P_j}\left| \right. {\dfrac{\partial U_j}{\partial \theta _x(j)} X_{j-1}}\right\rangle \\ Re \left\langle {P_j}\left| \right. {\dfrac{\partial U_j}{\partial \theta _y(j)} X_{j-1}}\right\rangle \end{pmatrix}\nonumber \\ & \quad \approx \begin{pmatrix}Re \left\langle {P_j}\left| \right. { - i \Delta t \, {\mathcal {H}}_{1_x}(j) \, X_j}\right\rangle \\ Re \left\langle {P_j}\left| \right. { - i \Delta t \, {{\mathcal {H}}_{1_y}(j)} \, X_j}\right\rangle \end{pmatrix} \end{aligned}$$

with $P_j = U^{\dagger }_{j+1} \cdots U^{\dagger }_{N} \ U_{\textrm{F}}$, $X_j = U_j \cdots U_1$ and previously used notations for the control Hamiltonian components ${\mathcal {H}}_{1_x}(j)$ and ${\mathcal {H}}_{1_y}(j)$.

### Analytical exact point-to-point gradients using x, y, and optional z controls

The usual equations for evolution of a spin density matrix have been used in the previous section. However, it might be advantageous to use the Liouville superoperator formalism for a single spin $\frac{1}{2}$ in Cartesian coordinate representation. The spin density operator can then be represented by a four vector $\rho = (\rho _{ \textbf{1}}, \rho _x, \rho _y, \rho _z)^T$, where the contribution of the identity matrix may be neglected. If furthermore relaxation is neglected, the spin density at time point *j* is fully represented by the vector

15 $$\begin{aligned} \rho _{\textrm{j}}= \begin{pmatrix} \rho _{\textrm{x}} \\ \rho _{\textrm{y}} \\ \rho _{\textrm{z}} \end{pmatrix} \end{aligned}$$

and propagation is achieved by the Liouville superoperators $R_j$ in the reduced Cartesian representation

16 $$\begin{aligned} \rho _{\textrm{j}}=R_{\textrm{j}}...R_{\textrm{1}}\rho _0 \end{aligned}$$

and the co-state is represented accordingly by

17 $$\begin{aligned} \lambda _j=R_{\textrm{j}}^{-1}...R_{\textrm{N}}^{-1}\lambda _{\textrm{F}}. \end{aligned}$$

Please note, that the spin density as well as the co-state vector only have to be multiplied single-sided by the corresponding Liouville superoperators. Since we are in the Cartesian component basis set, we furthermore see that the superoperator is represented by a simple rotation matrix, which can be written as

18 $$\begin{aligned} R_{\textrm{j}}= \begin{pmatrix} \cos (\theta )+n_x^2(1-\cos (\theta )) & -n_z\sin (\theta )+n_xn_y(1-\cos (\theta )) & n_y\sin (\theta )+n_xn_z(1-\cos (\theta )) \\ n_z\sin (\theta )+n_xn_y(1-\cos (\theta )) & \cos (\theta )+n_y^2(1-\cos (\theta )) & -n_x\sin (\theta )+n_yn_z(1-\cos (\theta )) \\ -n_y\sin (\theta )+n_xn_z(1-\cos (\theta )) & n_x\sin (\theta )+n_yn_z(1-\cos (\theta )) & \cos (\theta )+n_z^2(1-\cos (\theta )), \end{pmatrix} \end{aligned}$$

with the overall rotation angle $\theta$ and the normalized components $n_x$, $n_y$ and $n_z$ of the rotation axis of timestep *j*. It can also be noted more compact using the Rodrigues formula using its individual elements $R_{hk}$:

19 $$\begin{aligned} R_{hk}={\left\{ \begin{array}{ll} \cos ^{2}\bigg (\dfrac{\theta }{2}\bigg )+(2n_h^2-1)\sin ^{2}\bigg (\dfrac{\theta }{2}\bigg ) & \text {f}\ddot{\textrm{u}}\text {r}~h=k \\ 2n_hn_k\sin ^{2}\bigg (\dfrac{\theta }{2}\bigg )-\epsilon _{hkl}~n_l\sin (\theta ) & \text {f}\ddot{\textrm{u}}\text {r}~h\ne k, \end{array}\right. } \end{aligned}$$

where $\epsilon _{hkl}$ is the Levi-Civita symbol.

In each step *j*, $\theta$, $n_x$, $n_y$ and $n_z$ can be calculated from the control values $\omega _x$, $\omega _y$ and $\omega _z$ as follows:

20 $$\begin{aligned} \theta&=\Delta t\sqrt{\omega _x^2+\omega _y^2+(\omega _z+\omega _{\textrm{off}})^2} = \sqrt{\theta _x^2+\theta _y^2+\theta _z^2}\end{aligned}$$

21 $$\begin{aligned} n_x&=\dfrac{\omega _x}{\sqrt{\omega _x^2+\omega _y^2+(\omega _z+\omega _{\textrm{off}})^2}}=\dfrac{\theta _x}{\sqrt{\theta _x^2+\theta _y^2+\theta _z^2}} = \dfrac{\theta _x}{\theta }\end{aligned}$$

22 $$\begin{aligned} n_y&= \dfrac{\omega _y}{\sqrt{\omega _x^2+\omega _y^2+(\omega _z+\omega _{\textrm{off}})^2}} =\dfrac{\theta _y}{\sqrt{\theta _x^2+\theta _y^2+\theta _z^2}} = \dfrac{\theta _y}{\theta } \end{aligned}$$

23 $$\begin{aligned} n_z&= \dfrac{\omega _z+\omega _{\textrm{off}}}{\sqrt{\omega _x^2+\omega _y^2+(\omega _z+\omega _{\textrm{off}})^2}} =\dfrac{\theta _z+\theta _{\textrm{off}}}{\sqrt{\theta _x^2+\theta _y^2+\theta _z^2}} = \dfrac{\theta _z}{\theta }. \end{aligned}$$

with the effective flip angles around the Cartesian axes $\theta _x = \omega _x \Delta t$, $\theta _y = \omega _y \Delta t$, $\theta _z = (\omega _z + \omega _{\textrm{off}}) \Delta t$, where the rotation around the *z*-axis now consists of the offset term introduced above and the introduction of a potential *z*-control that cannot be applied directly on a spectrometer, but that can be used to allow direct implementation of pulse sequence bound offset changes in optimizations (Stockmann and Wald 2018; Vinding et al. 2017, 2021). With these equations in hand, it is straightforward to reformulate the cost function for point-to-point pulses in Cartesian Liouvielle representation as

24 $$\begin{aligned} & \Phi _{\textrm{PP}}=\left\langle {\lambda _{\textrm{F}}}\left| \right. {\rho _{\textrm{N}}}\right\rangle =\left\langle {\lambda _{\textrm{F}}}\left| \right. {R_{\textrm{N}}...R_{\textrm{1}}\rho _0}\right\rangle =\Big \langle R_{\mathrm {j+1}}^{-1}...R_{\textrm{n}}^{-1}\lambda _{\textrm{n}}\Big | R_{\textrm{j}}...R_{\textrm{1}}\rho _0\Big \rangle \nonumber \\ & \quad =\Big \langle \underbrace{R_{\mathrm {j+1}}^{T}...R_{\textrm{n}}^{T}\lambda _{\textrm{n}}}_{\textstyle \lambda _{\textrm{j}}}\Big | \underbrace{R_{\textrm{j}}...R_{\textrm{1}}\rho _0}_{\textstyle \rho _{\textrm{j}}}\Big \rangle . \end{aligned}$$

Accordingly, the gradients for $j^{th}$ time point for the single spin is

25 $$\begin{aligned} \Gamma _{\textrm{PP}}(j)=\begin{pmatrix}\dfrac{\partial \Phi _{\textrm{PP}}}{\partial \theta _x(j)} \\ \dfrac{\partial \Phi _{\textrm{PP}}}{\partial \theta _y(j)} \\ \dfrac{\partial \Phi _{\textrm{PP}}}{\partial \theta _z(j)} \end{pmatrix} = \begin{pmatrix}\left\langle {\lambda _j}\left| \right. { \dfrac{\partial R_j}{\partial \theta _x(j)} \rho _{j-1}}\right\rangle \\ \left\langle {\lambda _j}\left| \right. { \dfrac{\partial R_j}{\partial \theta _y(j)} \rho _{j-1}}\right\rangle \\ \left\langle {\lambda _j}\left| \right. { \dfrac{\partial R_j}{\partial \theta _z(j)} \rho _{j-1}}\right\rangle \end{pmatrix}, \end{aligned}$$

which leaves an analytical derivation of the derivative of the rotation matrix with respect to the Cartesian controls to obtain an overall exact gradient. With the formulae given in this section, the derivatives of the individual matrix components can be straightforwardly achieved and corresponding results are shown in Table 1 for the *x* and*y* derivatives and in Table 2 for the *z*-control.

**Table 1:** Derivatives of rotation matrix components with respect to $\theta _{x}$ and $\theta _{y}$ for point-to-point optimizations

$$\dfrac{\partial R_{xx}}{\partial \theta _x} = (n_x^3 - n_x)\Bigg ( \sin (\theta ) + \dfrac{2 \ (\cos (\theta )-1)}{\theta } \Bigg )$$	
$$\dfrac{\partial R_{xy}}{\partial \theta _x} =\dfrac{n_y-2 n_x^2n_y}{\theta } + \Bigg ( \dfrac{2 n_x^2n_y-n_y}{\theta } - n_xn_z \Bigg ) \cos (\theta ) + \Bigg ( n_x^2n_y +\dfrac{n_xn_z}{\theta } \Bigg ) \sin (\theta )$$	
$$\dfrac{\partial R_{xz}}{\partial \theta _x} =\dfrac{n_z-2 n_x^2n_z}{\theta } + \Bigg ( \dfrac{2 n_x^2n_z-n_z}{\theta } + n_xn_y \Bigg ) \cos (\theta ) + \Bigg ( n_x^2n_z -\dfrac{n_xn_y}{\theta } \Bigg ) \sin (\theta )$$	
$$\dfrac{\partial R_{yx}}{\partial \theta _x} =\dfrac{n_y-2 n_x^2n_y}{\theta } + \Bigg ( \dfrac{2 n_x^2n_y - n_y}{\theta } + n_xn_z \Bigg ) \cos (\theta ) + \Bigg ( n_x^2n_y -\dfrac{n_xn_z}{\theta } \Bigg ) \sin (\theta )$$	
$$\dfrac{\partial R_{yy}}{\partial \theta _x} =(n_xn_y^2-n_x)\sin (\theta )+n_xn_y^2 \ \Bigg (\dfrac{2 \ \left( \cos (\theta )-1\right) }{\theta }\Bigg )$$	
$$\dfrac{\partial R_{yz}}{\partial \theta _x} =- \dfrac{2 n_x n_y n_z}{\theta } + \Bigg ( \dfrac{2 n_xn_yn_z}{\theta } - n_x^2 \Bigg ) \cos (\theta ) + \Bigg ( n_xn_yn_z -\dfrac{(n_y^2+n_z^2)}{\theta } \Bigg ) \sin (\theta )$$	
$$\dfrac{\partial R_{zx}}{\partial \theta _x} =\dfrac{n_z-2 n_x^2n_z}{\theta } + \Bigg ( \dfrac{2 n_x^2n_z-n_z}{\theta } - n_xn_y \Bigg ) \cos (\theta ) + \Bigg ( n_x^2n_z +\dfrac{n_xn_y}{\theta } \Bigg ) \sin (\theta )$$	
$$\dfrac{\partial R_{zy}}{\partial \theta _x} =- \dfrac{2 n_x n_y n_z}{\theta } + \Bigg ( \dfrac{2 n_xn_yn_z}{\theta } + n_x^2 \Bigg ) \cos (\theta ) + \Bigg ( n_xn_yn_z +\dfrac{(n_y^2+n_z^2)}{\theta } \Bigg ) \sin (\theta )$$	
$$\dfrac{\partial R_{zz}}{\partial \theta _x} =(n_xn_z^2-n_x)\sin (\theta )+n_xn_z^2 \ \Bigg (\dfrac{2 \ \left( \cos (\theta )-1\right) }{\theta }\Bigg )$$	
$$\dfrac{\partial R_{xx}}{\partial \theta _y} =(n_yn_x^2-n_y)\sin (\theta )+n_yn_x^2 \ \Bigg (\dfrac{2 \ \left( \cos (\theta )-1\right) }{\theta }\Bigg )$$	
$$\dfrac{\partial R_{xy}}{\partial \theta _y} =\dfrac{n_x-2 n_y^2n_x}{\theta } + \Bigg ( \dfrac{2 n_y^2n_x-n_x}{\theta } - n_yn_z \Bigg ) \cos (\theta ) + \Bigg ( n_y^2n_x +\dfrac{n_yn_z}{\theta } \Bigg ) \sin (\theta )$$	
$$\dfrac{\partial R_{xz}}{\partial \theta _y} =- \dfrac{2 n_x n_y n_z}{\theta } + \Bigg ( \dfrac{2 n_xn_yn_z}{\theta } + n_y^2 \Bigg ) \cos (\theta ) + \Bigg ( n_xn_yn_z +\dfrac{(n_x^2+n_z^2)}{\theta } \Bigg ) \sin (\theta )$$	
$$\dfrac{\partial R_{yx}}{\partial \theta _y} =\dfrac{n_x-2 n_y^2n_x}{\theta } + \Bigg ( \dfrac{2 n_y^2n_x-n_x}{\theta } + n_yn_z \Bigg ) \cos (\theta ) + \Bigg ( n_y^2n_x -\dfrac{n_yn_z}{\theta } \Bigg ) \sin (\theta )$$	
$$\dfrac{\partial R_{yy}}{\partial \theta _y} = (n_y^3 - n_y)\Bigg ( \sin (\theta ) + \dfrac{2 \ (\cos (\theta )-1)}{\theta } \Bigg )$$	
$$\dfrac{\partial R_{yz}}{\partial \theta _y} =\dfrac{n_z-2 n_y^2n_z}{\theta } + \Bigg ( \dfrac{2 n_y^2n_z-n_z}{\theta } - n_xn_y \Bigg ) \cos (\theta ) + \Bigg ( n_y^2n_z +\dfrac{n_xn_y}{\theta } \Bigg ) \sin (\theta )$$	
$$\dfrac{\partial R_{zx}}{\partial \theta _y} =- \dfrac{2 n_x n_y n_z}{\theta } + \Bigg ( \dfrac{2 n_xn_yn_z}{\theta } - n_y^2 \Bigg ) \cos (\theta ) + \Bigg ( n_xn_yn_z -\dfrac{(n_x^2+n_z^2)}{\theta } \Bigg ) \sin (\theta )$$	
$$\dfrac{\partial R_{zy}}{\partial \theta _y} =\dfrac{n_z-2 n_y^2n_z}{\theta } + \Bigg ( \dfrac{2 n_y^2n_z-n_z}{\theta } + n_xn_y \Bigg ) \cos (\theta ) + \Bigg ( n_y^2n_z -\dfrac{n_xn_y}{\theta } \Bigg ) \sin (\theta )$$	
$$\dfrac{\partial R_{zz}}{\partial \theta _y} =(n_yn_z^2-n_y)\sin (\theta )+n_yn_z^2 \ \Bigg (\dfrac{2 \ \left( \cos (\theta )-1\right) }{\theta }\Bigg )$$

**Table 2:** Derivatives of rotation matrix components with respect to $\theta _{z}$ for point-to-point optimizations

$$\dfrac{\partial R_{xx}}{\partial \theta _z} =(n_zn_x^2-n_z)\sin (\theta )+n_zn_x^2 \ \Bigg (\dfrac{2 \ \left( \cos (\theta )-1\right) }{\theta }\Bigg )$$	
$$\dfrac{\partial R_{xy}}{\partial \theta _z} =- \dfrac{2 n_x n_y n_z}{\theta } + \Bigg ( \dfrac{2 n_xn_yn_z}{\theta } - n_z^2 \Bigg ) \cos (\theta ) + \Bigg ( n_xn_yn_z -\dfrac{(n_y^2+n_x^2)}{\theta } \Bigg ) \sin (\theta )$$	
$$\dfrac{\partial R_{xz}}{\partial \theta _z} =\dfrac{n_x-2 n_z^2n_x}{\theta } + \Bigg ( \dfrac{2 n_z^2n_x-n_x}{\theta } + n_zn_y \Bigg ) \cos (\theta ) + \Bigg ( n_z^2n_x -\dfrac{n_zn_y}{\theta } \Bigg ) \sin (\theta )$$	
$$\dfrac{\partial R_{yx}}{\partial \theta _z} =- \dfrac{2 n_x n_y n_z}{\theta } + \Bigg ( \dfrac{2 n_xn_yn_z}{\theta } + n_z^2 \Bigg ) \cos (\theta ) + \Bigg ( n_xn_yn_z +\dfrac{(n_y^2+n_x^2)}{\theta } \Bigg ) \sin (\theta )$$	
$$\dfrac{\partial R_{yy}}{\partial \theta _z} =(n_zn_y^2-n_z)\sin (\theta )+n_zn_y^2 \ \Bigg (\dfrac{2 \ \left( \cos (\theta )-1\right) }{\theta }\Bigg )$$	
$$\dfrac{\partial R_{yz}}{\partial \theta _z} =\dfrac{n_y-2 n_z^2n_y}{\theta } + \Bigg ( \dfrac{2 n_z^2n_y - n_y}{\theta } - n_xn_z \Bigg ) \cos (\theta ) + \Bigg ( n_z^2n_y +\dfrac{n_xn_z}{\theta } \Bigg ) \sin (\theta )$$	
$$\dfrac{\partial R_{zx}}{\partial \theta _z} =\dfrac{n_x-2 n_z^2n_x}{\theta } + \Bigg ( \dfrac{2 n_z^2n_x-n_x}{\theta } - n_zn_y \Bigg ) \cos (\theta ) + \Bigg ( n_z^2n_x +\dfrac{n_zn_y}{\theta } \Bigg ) \sin (\theta )$$	
$$\dfrac{\partial R_{zy}}{\partial \theta _z} =\dfrac{n_y-2 n_z^2n_y}{\theta } + \Bigg ( \dfrac{2 n_z^2n_y - n_y}{\theta } + n_xn_z \Bigg ) \cos (\theta ) + \Bigg ( n_z^2n_y -\dfrac{n_xn_z}{\theta } \Bigg ) \sin (\theta )$$	
$$\dfrac{\partial R_{zz}}{\partial \theta _z} = (n_z^3 - n_z)\Bigg ( \sin (\theta ) + \dfrac{2 \ (\cos (\theta )-1)}{\theta } \Bigg )$$	

### Analytical exact point-to-point gradients using amplitude, phase, and optional z controls

The normalized components of the rotation axes $n_x$, $n_y$ and $n_z$ can also be written in polar coordinates, resulting in the pulse phase $\alpha$ and rf-amplitude $\omega _{\textrm{rf}}$ as the input parameters for pulse shapes. The corresponding notation is

26 $$\begin{aligned} n_x= & \dfrac{\cos (\alpha )\theta _{xy}}{\theta }=\dfrac{\cos (\alpha )\omega _{\textrm{rf}}\Delta t}{\sqrt{(\Delta t^2\omega _{\textrm{rf}})^2+\theta _z^2}}, ~n_y =\dfrac{\sin (\alpha )\theta _{xy}}{\theta }\nonumber \\ & =\dfrac{\sin (\alpha )\omega _{\textrm{rf}}\Delta t}{\sqrt{(\Delta t^2\omega _{\textrm{rf}})^2+\theta _z^2}}, ~n_z =\dfrac{\theta _z}{\theta }=\dfrac{\theta _z}{\sqrt{(\Delta t^2\omega _{\textrm{rf}})^2+\theta _z^2}} \end{aligned}$$

with

27 $$\begin{aligned} \theta _{xy}&=\omega _{\textrm{rf}}\Delta t \nonumber \\ \theta _z&=(\omega _z+\omega _{\textrm{off}})\Delta t \nonumber \\ \theta&=\sqrt{\theta _{xy}^2 + \theta _z^2} \end{aligned}$$

The derivatives for the rotational matrix components with respect to $\alpha$, $\omega _{\textrm{rf}}$ and $\omega _z$ can then be derived the same way as in the previous section. The gradients for the $j^{th}$ time point for the single spin are

28 $$\begin{aligned} \Gamma _{\textrm{PP}}(j)=\begin{pmatrix}\dfrac{\partial \Phi _{\textrm{PP}}}{\partial \alpha (j)} \\ \dfrac{\partial \Phi _{\textrm{PP}}}{\partial \theta _{\textrm{xy}}(j)} \\ \dfrac{\partial \Phi _{\textrm{PP}}}{\partial \theta _z(j)} \end{pmatrix} = \begin{pmatrix}\left\langle {\lambda _j}\left| \right. { \dfrac{\partial R_j}{\partial \alpha (j)} \rho _{j-1}}\right\rangle \\ \left\langle {\lambda _j}\left| \right. { \dfrac{\partial R_j}{\partial \theta _{\textrm{xy}}(j)} \rho _{j-1}}\right\rangle \\ \left\langle {\lambda _j}\left| \right. { \dfrac{\partial R_j}{\partial \theta _z(j)} \rho _{j-1}}\right\rangle \end{pmatrix}, \end{aligned}$$

which again leaves an analytical derivation of the derivative of the rotation matrix with respect to the Cartesian controls to obtain an overall exact gradient. The calculation can be done by hand or a symbolic mathematics program and the result for the rotation matrix components are listed in Tables 3 and 4, respectively. The amplitude and phase representation is particularly useful in cases of restricted rf-amplitudes or even constant rf-amplitudes (Skinner et al. 2006), where in the latter case only derivatives to the phase $\alpha$ need to be considered for a minimum set of parameters.

**Table 3:** Derivatives of rotation matrix components with respect to phase $\alpha$ and rf-amplitude $\theta _{xy}$ for point-to-point optimizations

$$\dfrac{\partial R_{xx}}{\partial \alpha }=-2n_xn_y(1 - \cos (\theta ))$$	
$$\dfrac{\partial R_{xy}}{\partial \alpha }=(n_x^2 - n_y^2)(1 - \cos (\theta ))$$	
$$\dfrac{\partial R_{xz}}{\partial \alpha }=-n_yn_z(1 - \cos (\theta )) + n_x\sin (\theta )$$	
$$\dfrac{\partial R_{yx}}{\partial \alpha }=(n_x^2 - n_y^2)(1 - \cos (\theta ))$$	
$$\dfrac{\partial R_{yy}}{\partial \alpha }=2n_xn_y(1 - \cos (\theta ))$$	
$$\dfrac{\partial R_{yz}}{\partial \alpha }=n_xn_z(1 - \cos (\theta )) + n_y\sin (\theta )$$	
$$\dfrac{\partial R_{zx}}{\partial \alpha }=-n_yn_z(1 - \cos (\theta )) - n_x\sin (\theta )$$	
$$\dfrac{\partial R_{zy}}{\partial \alpha }=n_xn_z(1 - \cos (\theta )) - n_y\sin (\theta )$$	
$$\dfrac{\partial R_{zz}}{\partial \alpha }=0$$	
$$\dfrac{\partial R_{xx}}{\partial \theta _{xy}}=-\dfrac{\theta _{xy}(n_y^2+n_z^2)}{\theta }\sin (\theta )+\dfrac{2n_x^2n_z^2}{\theta _{xy}}(1-\cos (\theta ))$$	
$$\dfrac{\partial R_{xy}}{\partial \theta _{xy}}=-n_{xy} n_{z}\left( \cos (\theta ) - \dfrac{\sin (\theta )}{\theta }\right) + n_{x} n_{y} n_{xy} \sin (\theta ) + 2 \left( \dfrac{n_{x} n_{y}}{\theta _{xy}} - \dfrac{n_{x} n_{y} n_{xy}}{\theta }\right) (1 - \cos (\theta ))$$	
$$\dfrac{\partial R_{xz}}{\partial \theta _{xy}}=\left( \dfrac{n_{y}}{\theta _{xy}} - \dfrac{n_{y} n_{xy}}{\theta } + n_{x} n_{z} n_{xy}\right) \sin (\theta ) + n_{y} n_{xy} \cos (\theta ) + \left( \dfrac{n_{x} n_{z}}{\theta _{xy}} - \dfrac{2 n_{x} n_{z} n_{xy}}{\theta }\right) (1 - \cos (\theta ))$$	
$$\dfrac{\partial R_{yx}}{\partial \theta _{xy}}=n_{xy} n_{z}\left( \cos (\theta ) - \dfrac{\sin (\theta )}{\theta }\right) + n_{x} n_{y} n_{xy} \sin (\theta ) + 2 \left( \dfrac{n_{x} n_{y}}{t_{xy}} - \dfrac{n_{x} n_{y} n_{xy}}{\theta }\right) (1 - \cos (\theta ))$$	
$$\dfrac{\partial R_{yy}}{\partial \theta _{xy}}=-\dfrac{\theta _{xy}(n_x^2+n_z^2)}{\theta }\sin (\theta )+\dfrac{2n_y^2n_z^2}{\theta _{xy}}(1-\cos (\theta ))$$	
$$\dfrac{\partial R_{yz}}{\partial \theta _{xy}}=\left( -\dfrac{n_{x}}{\theta _{xy}} + \dfrac{n_{x} n_{xy}}{\theta } + n_{y} n_{z} n_{xy}\right) \sin (\theta ) - n_{x} n_{xy} \cos (\theta ) + \left( \dfrac{n_{y} n_{z}}{\theta _{xy}} - \dfrac{2 n_{y} n_{z} n_{xy}}{\theta }\right) (1 - \cos (\theta ))$$	
$$\dfrac{\partial R_{zx}}{\partial \theta _{xy}}=\left( -\dfrac{n_{y}}{\theta _{xy}} + \dfrac{n_{y} n_{xy}}{\theta } + n_{x} n_{z} n_{xy}\right) \sin (\theta ) - n_{y} n_{xy} \cos (\theta ) + \left( \dfrac{n_{x} n_{z}}{\theta _{xy}} - \dfrac{2 n_{x} n_{z} n_{xy}}{\theta }\right) (1 - \cos (\theta ))$$	
$$\dfrac{\partial R_{zy}}{\partial \theta _{xy}}=\left( \dfrac{n_{x}}{\theta _{xy}} - \dfrac{n_{x} n_{xy}}{\theta } + n_{y} n_{z} n_{xy}\right) \sin (\theta ) + n_{x} n_{xy} \cos (\theta ) + \left( \dfrac{n_{y} n_{z}}{\theta _{xy}} - \dfrac{2 n_{y} n_{z} n_{xy}}{\theta }\right) (1 - \cos (\theta ))$$	
$$\dfrac{\partial R_{zz}}{\partial \theta _{xy}}=-2n_{xy}n_{z}^2\dfrac{(1-\cos (\theta ))}{\theta }-n_{xy}^3(n_{xy}^2+n_{z}^2) \sin (\theta )$$

**Table 4:** Derivatives of rotation matrix components with respect to *z* rotations in polar coordinates of the *xy*-plane for point-to-point optimizations

$$\dfrac{\partial R_{xx}}{\partial \theta _{z}}=(n_x^2 n_z - n_z)\sin (\theta ) - \dfrac{2 n_x^2 n_z}{\theta }(1-\cos (\theta ))$$	
$$\dfrac{\partial R_{xy}}{\partial \theta _{z}}= \Bigg (n_x n_y n_z - \dfrac{\theta _{xy}^2}{\theta ^3}\Bigg ) \sin (\theta ) + \dfrac{2 n_x n_y n_z}{\theta }(\cos (\theta )-1) - n_z^2 \cos (\theta )$$	
$$\dfrac{\partial R_{xz}}{\partial \theta _{z}}= \Bigg (n_x n_z^2 - \dfrac{n_y n_z}{\theta } \Bigg ) \sin (\theta ) + \dfrac{(2 n_x n_z^2 - n_x)}{\theta } (\cos (\theta )-1) + n_y n_z \cos (\theta )$$	
$$\dfrac{\partial R_{yx}}{\partial \theta _{z}}= \Bigg (n_x n_y n_z + \dfrac{\theta _{xy}^2}{\theta ^3} \Bigg ) \sin (\theta ) + \dfrac{2 n_x n_y n_z}{\theta }(\cos (\theta )-1) + n_z^2 \cos (\theta )$$	
$$\dfrac{\partial R_{yy}}{\partial \theta _{z}}=(n_y^2 n_z - n_z)\sin (\theta ) - \dfrac{2 n_y^2 n_z}{\theta }(1-\cos (\theta ))$$	
$$\dfrac{\partial R_{yz}}{\partial \theta _{z}}= \Bigg (n_y n_z^2 + \dfrac{n_x n_z}{\theta } \Bigg )\sin (\theta ) + \dfrac{(2 n_y n_z^2 - n_y)}{\theta } (\cos (\theta )-1) - n_x n_z \cos (\theta )$$	
$$\dfrac{\partial R_{zx}}{\partial \theta _{z}}= \Bigg (n_x n_z^2 + \dfrac{n_y n_z}{\theta } \Bigg )\sin (\theta ) + \dfrac{(2 n_x n_z^2 - n_x)}{\theta } (\cos (\theta )-1) - n_y n_z \cos (\theta )$$	
$$\dfrac{\partial R_{zy}}{\partial \theta _{z}}= \Bigg (n_y n_z^2 - \dfrac{n_x n_z}{\theta } \Bigg )\sin (\theta ) + \dfrac{(2 n_y n_z^2 - n_y)}{\theta } (\cos (\theta )-1) + n_x n_z \cos (\theta )$$	
$$\dfrac{\partial R_{zz}}{\partial \theta _{z}}=(n_z^3 - n_z)\sin (\theta ) + \dfrac{2(n_z - n_z^3 )}{\theta }(1-\cos (\theta ))$$	

### Analytical exact universal rotation gradients

We have seen that propagation can be expressed in terms of simple rotations in the case of a single spin $\frac{1}{2}$. If effective rotations themselves need to be optimized, it is best to express them with minimum storage and computation time. As such, Cayley (1997) or, equivalently, quaternions (Emsley and Bodenhausen 1990; Hamilton 1843; Rodrigues 1840; Slad et al. 2022) can be used to express rotations. Although the minimum set of numbers to represent a rotation is the vector spanned by $(\theta _x, \theta _y, \theta _z)$, it is better to use a four-dimensional vector

29 $$\begin{aligned} Q_j = \begin{pmatrix} A_j \\ B_j\\ C_j \\ D_j \end{pmatrix} \end{aligned}$$

for the rotation at time step *j*, where the four components are defined by

30 $$\begin{aligned} A_{{j}}&= n_x(j) \ \sin (\frac{\theta (j)}{2}),\end{aligned}$$

31 $$\begin{aligned} B_{{j}}&= n_y(j) \ \sin (\frac{\theta (j)}{2}),\end{aligned}$$

32 $$\begin{aligned} C_{{j}}&= n_z(j) \ \sin (\frac{\theta (j)}{2}),\end{aligned}$$

33 $$\begin{aligned} D_{{j}}&= \cos (\frac{\theta (j)}{2}) \end{aligned}$$

using the definitions of Eqs. (20)-(22). Quaternions have the advantage that a series of rotations can be directly evaluated via the product

34 $$\begin{aligned} {Q_2} \cdot {Q}_{1}= \begin{pmatrix} +{D_2} & -{C_2} & +{B_2} & +A_2 \\ +{C_2} & +{B_2} & -A_2 & +{B_2} \\ -{B_2} & +A_2 & +{D_2} & +{C_2} \\ -A_2 & -{B_2} & -{C_2} & +{D_2} \end{pmatrix}\begin{pmatrix} {A}_{1} \\ {B}_{1} \\ {C}_{1} \\ {D}_{1} \end{pmatrix} \end{aligned}$$

which, with its 16 simple multiplications and sums, is computationally more efficient than the construction of a rotation matrix involving at least one sine/cosine calculation.

Using the formalism for universal rotation pulses, the cost function can be written directly as

35 $$\begin{aligned} \Phi _{\textrm{UR}}=Re\left\langle {U_{\textrm{F}}}\left| \right. {U_N}\right\rangle =Re\left\langle {Q_{\textrm{F}}}\left| \right. {Q_N \cdots Q_j \cdots Q_1}\right\rangle \end{aligned}$$

and the gradients are derived as

36 $$\begin{aligned} \Gamma _{\textrm{UR}}(j)=\begin{pmatrix}\dfrac{\partial \Phi _{\textrm{UR}}}{\partial \theta _x(j)} \\ \dfrac{\partial \Phi _{\textrm{UR}}}{\partial \theta _y(j)} \\ \dfrac{\partial \Phi _{\textrm{UR}}}{\partial \theta _z(j)} \end{pmatrix} = \begin{pmatrix}Re \left\langle {Q_{\textrm{F}}}\left| \right. {Q_N \cdots Q_{j+1} \cdot \dfrac{\partial Q_j}{\partial \theta _x(j)} \cdot Q_{j-1} \cdots Q_1 }\right\rangle \\ Re \left\langle {Q_{\textrm{F}}}\left| \right. {Q_N \cdots Q_{j+1} \cdot \dfrac{\partial Q_j}{\partial \theta _y(j)} \cdot Q_{j-1} \cdots Q_1 }\right\rangle \\ Re \left\langle {Q_{\textrm{F}}}\left| \right. {Q_N \cdots Q_{j+1} \cdot \dfrac{\partial Q_j}{\partial \theta _z(j)} \cdot Q_{j-1} \cdots Q_1 }\right\rangle \end{pmatrix}, \end{aligned}$$

where again the overall gradient calculation is reduced to simple quaternion propagation plus the calculation of the rotation derivative - this time in quaternion notation. With Eqs. (30)-(33) the involved partial derivatives of the individual quaternion components can be calculated and corresponding results are summarized in Table 5. Obviously, the components of interest can also be derived with respect to polar coordinates $\alpha$ and $\theta _{xy}$ in the *xy*-plane, for which the terms are given in the right column of the same Table. It should be noted that the derivatives with respect to $\alpha$ are particularly simple, which guarantees fastest gradient calculation times for the case of constant amplitude pulses.

**Table 5:** Exact gradients of the quaternion elements *A*, *B*, *C*, *D* with respect to cartesian coordinates ($\theta _x$, $\theta _y$ and $\theta _z$) and polar coordinates ($\alpha$,$\theta _{xy}$ and $\theta _z$). Auxiliary variable is $n_{xy} = \frac{\theta {xy}}{\theta }$, other variables as defined in the main text

Cartesian		Polar	
$\dfrac{\partial A}{\partial \theta _x}=$	$(1-n_{x}^2) \ \dfrac{\sin (\theta /2)}{\theta }+n_{x}^2 \ \dfrac{\cos (\theta /2)}{2}$	$\dfrac{\partial A}{\partial \alpha }=$	$- \theta _{xy}\sin (\alpha ) \ \dfrac{\sin (\theta /2)}{\theta }$
$\dfrac{\partial B}{\partial \theta _x}=$	$n_{x}n_y\bigg (\dfrac{\cos (\theta /2)}{2}-\dfrac{\sin (\theta /2)}{\theta }\bigg )$	$\dfrac{\partial B}{\partial \alpha }=$	$\ \ \theta _{xy}\cos (\alpha ) \ \dfrac{\sin (\theta /2)}{\theta }$
$\dfrac{\partial C}{\partial \theta _x}=$	$n_{x}n_z\bigg (\dfrac{\cos (\theta /2)}{2}-\dfrac{\sin (\theta /2)}{\theta }\bigg )$	$\dfrac{\partial C}{\partial \alpha }=$	0
$\dfrac{\partial D}{\partial \theta _x}=$	$-n_{x} \ \dfrac{\sin (\theta /2)}{2}$	$\dfrac{\partial D}{\partial \alpha }=$	0
$\dfrac{\partial A}{\partial \theta _y}=$	$n_{x}n_y\bigg (\dfrac{\cos (\theta /2)}{2}-\dfrac{\sin (\theta /2)}{\theta }\bigg )$	$\dfrac{\partial A}{\partial \theta _{xy}}=$	$\cos (\alpha )\bigg ( (1-n_{xy}^2) \ \dfrac{\sin (\theta /2)}{\theta }+n_{xy}^2 \ \dfrac{\cos (\theta /2)}{2}\bigg )$
$\dfrac{\partial B}{\partial \theta _y}=$	$(1-n_{y}^2) \ \dfrac{\sin (\theta /2)}{\theta }+n_{y}^2 \ \dfrac{\cos (\theta /2)}{2}$	$\dfrac{\partial B}{\partial \theta _{xy}}=$	$\sin (\alpha )\bigg ( (1-n_{xy}^2) \ \dfrac{\sin (\theta /2)}{\theta }+n_{xy}^2 \ \dfrac{\cos (\theta /2)}{2}\bigg )$
$\dfrac{\partial C}{\partial \theta _y}=$	$n_{y}n_z\bigg (\dfrac{\cos (\theta /2)}{2}-\dfrac{\sin (\theta /2)}{\theta }\bigg )$	$\dfrac{\partial C}{\partial \theta _{xy}}=$	$n_{xy}n_z\bigg (\dfrac{\cos (\theta /2)}{2}-\dfrac{\sin (\theta /2)}{\theta }\bigg )$
$\dfrac{\partial D}{\partial \theta _y}=$	$-n_{y} \ \dfrac{\sin (\theta /2)}{2}$	$\dfrac{\partial D}{\partial \theta _{xy}}=$	$-n_{xy} \ \dfrac{\sin (\theta /2)}{2}$
$\dfrac{\partial A}{\partial \theta _z}=$	$n_{x}n_z\bigg (\dfrac{\cos (\theta /2)}{2}-\dfrac{\sin (\theta /2)}{\theta }\bigg )$	$\dfrac{\partial A}{\partial \theta _{z}}=$	$n_{xy}n_z\cos (\alpha )\bigg (\dfrac{\cos (\theta /2)}{2}-\dfrac{\sin (\theta /2)}{\theta }\bigg )$
$\dfrac{\partial B}{\partial \theta _z}=$	$n_{y}n_z\bigg (\dfrac{\cos (\theta /2)}{2}-\dfrac{\sin (\theta /2)}{\theta }\bigg )$	$\dfrac{\partial B}{\partial \theta _{z}}=$	$n_{xy}n_z\sin (\alpha )\bigg (\dfrac{\cos (\theta /2)}{2}-\dfrac{\sin (\theta /2)}{\theta }\bigg )$
$\dfrac{\partial C}{\partial \theta _z}=$	$(1-n_z^2) \ \dfrac{\sin (\theta /2)}{\theta }+n_z^2 \ \dfrac{\cos (\theta /2)}{2}$	$\dfrac{\partial C}{\partial \theta _{z}}=$	$(1-n_z^2) \ \dfrac{\sin (\theta /2)}{\theta }+n_z^2 \ \dfrac{\cos (\theta /2)}{2}$
$\dfrac{\partial D}{\partial \theta _z}=$	$-n_z \ \dfrac{\sin (\theta /2)}{2}$	$\dfrac{\partial D}{\partial \theta _{z}}=$	$-n_z \ \dfrac{\sin (\theta /2)}{2}$

### Limited rf-amplitudes as holonomic constraints

Optimizing efficient pulses in magnetic resonance usually implies optimizations with boundary conditions. One of the fundamental boundaries concern large rf-amplitudes that can cause experimental problems in the form of arching, violated duty cycles, and exceedance of allowed energy depositions. As such, they pose clear restrictions to applicable rf-amplitudes, rf-power, or rf-energy, respectively. Simple implementations with hard cut-off limits have been proposed early on in optimal control optimizations (Gershenzon et al. 2008, 2007; Kobzar et al. 2004, 2008; Skinner et al. 2004). However, mathematically more sound are so-called holonomic constraints that can be included in optimizations as Lagrange multipliers. Holonomic constraints essentially require continuously differentiable functions for restrictions and are usually implemented by auxiliary variables. A multitude of periodic functions have been proposed as auxiliary functions (Goodwin 2017), but we will concentrate here on a widely used trigonometric function, the hyperbolic tangent $\tanh$, which can be incorporated as a reduced rf-amplitude in polar coordinate systems according to

37 $$\begin{aligned} \theta _{xy}^{\textrm{red}}(j) = \theta ^{\textrm{max}}(j) \ \cdot \ \tanh \left( \frac{\theta _{xy}(j)}{\theta ^{\textrm{max}}(j)} \right) \end{aligned}$$

where the reduced amplitude $\theta _{xy}^{\textrm{red}}(j)$ of the $j^{th}$ digit continuously converges only to the maximum value $\theta ^{\textrm{max}}(j)$. In an optimization, $\theta _{xy}(j)$ instead can now be changed as an auxiliary variable without any restriction, while $\theta _{xy}^{\textrm{red}}(j)$ will be used for the actual values of rf-amplitudes in the pulse shape.

For a simple amplitude restriction to a *j*-dependent maximum value,

38 $$\begin{aligned} \theta ^{\textrm{max}}(j) = \textrm{const} (j) = 2 \pi \ \Delta t (j) \ \nu _{\textrm{rf}}^{\textrm{max}}(j) \end{aligned}$$

with maximum rf-amplitude $\nu _{\textrm{rf}}^{\textrm{max}}(j)$ can be imposed. Usually a constant restriction for all time steps is chosen, but in special cases, physics may imply a *j*-dependent upper limit function (Skinner et al. 2012). In an actual optimization, we restrict ourselves to the polar case where the rf-amplitude is inherently included in the variable $\theta _{xy}$. However, using the auxiliary approach, the variables $n_x$, $n_y$, $n_z$, $\theta _{xy}$, and $\theta$ from equations (18,19,26,27,29 - 33) used in the rotation matrices as well as in the quaternion formalism, have to be replaced by their reduced variants according to

39 $$\begin{aligned} \theta _{xy}^{\textrm{red}}(j)= & \theta ^{\textrm{max}}(j) \ \cdot \ \tanh \left( \frac{\theta _{xy}(j)}{\theta ^{\textrm{max}}(j)} \right) \end{aligned}$$

40 $$\begin{aligned} \theta ^{\textrm{red}}(j)= & \sqrt{(\theta _{xy}^{\textrm{red}}(j))^2 + \theta _z(j)^2} \end{aligned}$$

41 $$\begin{aligned} n_x^{\textrm{red}}(j)= & \frac{\cos (\alpha ) \cdot \theta _{xy}^{\textrm{red}}(j)}{\theta ^{\textrm{red}}(j)} \end{aligned}$$

42 $$\begin{aligned} n_y^{\textrm{red}}(j)= & \frac{\sin (\alpha ) \cdot \theta _{xy}^{\textrm{red}}(j)}{\theta ^{\textrm{red}}(j)} \end{aligned}$$

43 $$\begin{aligned} n_x^{\textrm{red}}(j)= & \frac{\theta _{z}(j)}{\theta ^{\textrm{red}}(j)} \end{aligned}$$

The resulting gradients $\Gamma _{\textrm{PP}}$ and $\Gamma _{\textrm{UR}}$ are then essentially of the same form as derived above. Only for the derivation to the auxiliary variable itself the $\tanh$ is not a constant and care has to be taken. Taking the derivation of the $j^{th}$ element of an UR optimization as an example, we can derive

44 $$\begin{aligned} \frac{\partial \Phi _{\textrm{UR}}}{\partial \theta _{xy} (j)} = \underbrace{ \frac{\partial \Phi _{\textrm{UR}}}{\partial \theta _{xy}^{\textrm{red}} (j)}}_{\text {control}} \, \cdot \, \underbrace{ \frac{d \theta _{xy}^{\textrm{red}} (j)}{d \theta _{xy} (j)}}_{\text {auxiliary}} \end{aligned}$$

With the auxiliary derivative term the actual control $\theta _{xy}^{\textrm{red}} (j)$ (i.e. the rf-amplitudes of the shaped pulse) can be used, for the optimization, instead, the variable $\theta _{xy}$ has to be used, making it necessary to include the terms

45 $$\begin{aligned} \frac{d \theta _{xy}^{\textrm{red}} (j)}{d \theta _{xy} (j)} = \text {sech}^2 \left( \frac{\theta _{xy}(j)}{\theta ^{\textrm{max}}(j)} \right) = 1 - \tanh ^2 \left( \frac{\theta _{xy}(j)}{\theta ^{\textrm{max}}(j)} \right) \end{aligned}$$

into the equations of the gradient in question. Please note that the second solution is derived from the general relation $\text {sech}^2(x) + \tanh ^2(x) = 1$ and results in a computationally friendly term, as $\tanh \left( \frac{\theta _{xy}(j)}{\theta ^{\textrm{max}}(j)} \right)$ has to be calculated anyway.

If overall rf-power restrictions have to be imposed, the maximum rotation angle can also be defined via the reduced angle at time point *j* according to

46 $$\begin{aligned} \theta _{xy}^{\textrm{red}}(j) = \theta _{xy}(j) \ \cdot \ \sqrt{\frac{\overline{P^{\textrm{max}}}}{\overline{P}}} \ \cdot \ \tanh \left( \sqrt{\frac{\overline{P}}{\overline{P^{\textrm{max}}}}} \right) \end{aligned}$$

with the squareroots of the maximum allowed average rf-power $\overline{P^{\textrm{max}}}$ and actual average rf-power expressed by

47 $$\begin{aligned} \overline{P} = \sum _{j = 1}^N \frac{(\theta _{xy}(j))^2}{N} \end{aligned}$$

where piecewise constant rf-amplitudes and uniform time steps $\Delta t$ have been assumed. Again, the derivative of the reduced rotation angle has to be calculated for the gradient, but this time the reduction involves rotation angles at all time steps and the derivative in e.g. an UR optimization with rf-power restriction will involve

48 $$\begin{aligned} \frac{\partial \Phi _{\textrm{UR}}}{\partial \theta _{xy} (j)} = \sum _{k = 1}^N \ \frac{\partial \Phi _{\textrm{UR}}}{\partial \theta _{xy}^{\textrm{red}} (k)} \, \cdot \, \frac{\partial \theta _{xy}^{\textrm{red}} (k)}{\partial \theta _{xy} (j)} \end{aligned}$$

with the reduced rotation angle derivatives defined by

49 $$\begin{aligned} \frac{\partial \theta _{xy}^{\textrm{red}}(j)}{\partial \theta _{xy} (j)}= & \left( 1 - \frac{(\theta _{xy}(j))^2}{\sum _{i = 1}^N (\theta _{xy}(j))^2} \right) \cdot \sqrt{\frac{\overline{P^{\textrm{max}}}}{\overline{P}}} \tanh \left( \sqrt{\frac{\overline{P}}{\overline{P^{\textrm{max}}}}} \right) \nonumber \\ & + \frac{(\theta _{xy}(j))^2}{\sum _{i = 1}^N (\theta _{xy}(j))^2} \left( 1 - \tanh ^2 \left( \sqrt{\frac{\overline{P}}{\overline{P^{\textrm{max}}}}} \right) \right) \ \ \ \ \forall \ \ \ k=j \;\end{aligned}$$

50 $$\begin{aligned} \frac{\partial \theta _{xy}^{\textrm{red}}(k)}{\partial \theta _{xy} (j)}= & \frac{(\theta _{xy}(j))^2}{\sum _{i = 1}^N (\theta _{xy}(j))^2} \cdot \sqrt{\frac{\overline{P^{\textrm{max}}}}{\overline{P}}} \tanh \left( \sqrt{\frac{\overline{P}}{\overline{P^{\textrm{max}}}}} \right) \nonumber \\ & + \frac{(\theta _{xy}(j))^2}{\sum _{i = 1}^N (\theta _{xy}(j))^2} \left( 1 - \tanh ^2 \left( \sqrt{\frac{\overline{P}}{\overline{P^{\textrm{max}}}}} \right) \right) \ \ \ \ \forall \ \ \ k \ne j \ . \end{aligned}$$

Finally, also the overall rf-energy of a pulse can be restricted, using, for example, the energy defined in Hz

51 $$\begin{aligned} \frac{E}{h} = \overline{P} \, t_p, \end{aligned}$$

or the more convenient expression in terms of rotation angles

52 $$\begin{aligned} E_{\theta } = \sum _{i = 1}^N (\theta _{xy}(i))^2 = (2 \pi )^2 \ \Delta t \ \frac{E}{h} \end{aligned}$$

when equations (46 - 50) are used with substituting $\overline{P^{\textrm{max}}}$ with the maximum allowed energy $E_{\theta }^{\textrm{max}} = (2 \pi )^2 \Delta t E^{\textrm{max}} / h$ and $\overline{P}$ by $E_{\theta }$.

## Results and Discussion

### Runtime comparisons

All analytical solutions for the different gradients have been derived to significantly enhance computational performance. As the runtime performance of already existing optimization programs depends on a large number of different parameters which do not allow a fair comparison. Furthermore, some programs are limited to certain types of pulse shapes (SEEDLESS, for example, is fast, but also limited to constant amplitude pulses with exclusive optimization of pulse phases and does not include other options). All of the existing programs may, nevertheless, benefit from the analytical solutions derived here. We therefore implemented the analytical solutions in Julia, a modern compiling programming language with a comfortable interface similar to Python or Matlab. The implementation of linear algebra routines in Julia is furthermore based on efficient BLAS routines, that still represent today’s standard. We compared the analytical solutions derived in the various Tables with the exact solution provided by the augmented matrix exponential (Floether et al. 2012; Goodwin and Kuprov 2015; Van Loan 1978) approach and, in addition, to a finite difference approximation. All calculations were performed with double precision (Float64) accuracy.

For the Cartesian point-to-point case, the augmented matrix exponential approach for a specific Cartesian control can be implemented in a 6-dimensional matrix that consists of two identical rotation matrices along the diagonal, and the generator of corresponding the *x*, *y*, or *z*-control in the right upper corner. Solving the equation

53 $$\begin{aligned} & \begin{pmatrix} R_j & \frac{\partial R_j}{\partial \theta _{\alpha }(j)} \\ 0 & R_j \end{pmatrix} = \exp \begin{pmatrix} -i L \theta (j) & -i G_{\alpha } \Delta t \\ 0 & -i L \theta (j) \end{pmatrix}\nonumber \\ & \quad \ \ \ \textrm{with} \ \ \ L \theta (j) = (G_x \theta _x(j) + G_y \theta _y(j) +G_z \theta _z(j)) \ \ \ , \ \ \ \alpha \in \{x, y, z\} \end{aligned}$$

and

54 $$\begin{aligned} G_x = \begin{pmatrix} 0 & 0 & 0 \\ 0 & 0 & 1 \\ 0 & 1 & 0 \end{pmatrix} \ \ \ , \ \ \ G_y = \begin{pmatrix} 0 & 0 & 1 \\ 0 & 0 & 0 \\ 1 & 0 & 0 \end{pmatrix} \ \ \ , \ \ \ G_z = \begin{pmatrix} 0 & 1 & 0 \\ 1 & 0 & 0 \\ 0 & 0 & 0 \end{pmatrix} \end{aligned}$$

gives the desired result for the first derivative $\partial R_j / \partial \theta _{\alpha }$ in place of the control. It should be noted, that the augmented matrix exponential approach cannot directly be applied to polar coordinates, as no generator is available for a change of the phase-angle $\alpha$ or the overall rotation angle $\theta _{{xy}}$. This would imply the calculation of an x- and y-control and then combine the two results for obtaining phase and amplitude. We did not attempt this, as this clearly would result in doubling of calculation time and an even larger gap in performance of this approach. Correspondingly, the auxiliary $\tanh$ approach was not attempted, as it only makes sense in polar coordinates.

Equivalent to rotational matrices, the augmented matrix exponential approach can be applied using a single spin propagator defined via the Pauli matrices. The corresponding equation involves $4 \times 4$ matrices according to

55 $$\begin{aligned} & \begin{pmatrix} U_j & \frac{\partial U_j}{\partial \theta _{\alpha }(j)} \\ 0 & U_j \end{pmatrix} = \exp \begin{pmatrix} -i H \theta (j) & -i \sigma _{\alpha } \Delta t \\ 0 & -i H \theta (j) \end{pmatrix} \nonumber \\ & \quad \ \ \ \textrm{with} \ \ \ \sigma \theta (j) = (\sigma _x \theta _x(j) + \sigma _y \theta _y(j) +\sigma _z \theta _z(j)) \ \ \ , \ \ \ \alpha \in \{x, y, z\} \end{aligned}$$

and

56 $$\begin{aligned} \sigma _x = \begin{pmatrix} 0 & \frac{1}{2} \\ \frac{1}{2} & 0 \end{pmatrix} \ \ \ , \ \ \ \sigma _y = \begin{pmatrix} 0 & -\frac{i}{2} \\ \frac{i}{2} & 0 \end{pmatrix} \ \ \ , \ \ \ \sigma _z = \begin{pmatrix} \frac{1}{2} & 0 \\ 0 & -\frac{1}{2} \end{pmatrix}. \end{aligned}$$

Finite difference approximations were performed using 3D rotation matrices and quaternions in the PP and UR cases, respectively. They essentially consist of two rotations and the corresponding calculations are mainly determined by the computation of trigonometric terms. Averaged computation times were calculated using the Julia package BenchmarkTools. The resulting times averaged over 1000 derivative calculations are summarized in Table 6. In all cases the analytical derivative solutions introduced here outperforms the other approaches. Compared to the other exact calculations based on the augmented matrix exponential approach, improvements of factors 100–150 are observed. The finite differences approach, on the other hand, is quite fast with factors of approximately 1–3 for the different types of optimization. The gain of the analytical solution compared in this case is not so much in the speed, but, of course, in the higher accuracy of the gradient and therefore a faster overall convergence of the gradient-based optimizations.

**Table 6:** Benchmark runtimes of 1000 exact gradient calculations using the different formulae from Tables 1-7

PP (Cartesian)	Laptop$^a$	Workstation$^b$	UR (Cartesian)	Laptop$^a$	Workstation$^b$
Analytical			Analytical		
$\partial R / \partial \theta _x$	325.9$\mu$s	368.0$\mu$s	$\partial Q / \partial \theta _x$	271.4$\mu$s	331.3$\mu$s
$\partial R / \partial \theta _y$	298.2$\mu$s	361.7$\mu$s	$\partial Q / \partial \theta _y$	278.4$\mu$s	329.7$\mu$s
$\partial R / \partial \theta _z$	326.0$\mu$s	362.6$\mu$s	$\partial Q / \partial \theta _z$	288.7$\mu$s	333.4$\mu$s
Exponential			Exponential		
$\partial R / \partial \theta _x$	45788.0$\mu$s	33467.0$\mu$s	$\partial Q / \partial \theta _x$	43774.0$\mu$s	32058.0$\mu$s
$\partial R / \partial \theta _y$	49442.0$\mu$s	33990.0$\mu$s	$\partial Q / \partial \theta _y$	49276.0$\mu$s	32492.0$\mu$s
$\partial R / \partial \theta _z$	51196.0$\mu$s	33152.0$\mu$s	$\partial Q / \partial \theta _z$	48715.0$\mu$s	32651.0$\mu$s
Finite differences			Finite differences		
$\partial R / \partial \theta _x$	490.8$\mu$s	915.5$\mu$s	$\partial Q / \partial \theta _x$	311.3$\mu$s	328.9$\mu$s
$\partial R / \partial \theta _y$	505.4$\mu$s	907.5$\mu$s	$\partial Q / \partial \theta _y$	302.7$\mu$s	325.3$\mu$s
$\partial R / \partial \theta _z$	501.5$\mu$s	908.0$\mu$s	$\partial Q / \partial \theta _z$	307.3$\mu$s	325.3$\mu$s
PP (polar)			UR (polar)		
analytical			analytical		
$\partial R / \partial \alpha$	286.6$\mu$s	337.4$\mu$s	$\partial R / \partial \alpha$	287.4$\mu$s	330.2$\mu$s
$\partial R / \partial \theta _{xy}$	293.5$\mu$s	349.2$\mu$s	$\partial R / \partial \theta _{xy}$	281.9$\mu$s	354.0$\mu$s
$\partial R / \partial \theta _z$	276.1$\mu$s	335.5$\mu$s	$\partial R / \partial \theta _{z}$	277.0$\mu$s	332.5$\mu$s
Finite differences			Finite differences		
$\partial R / \partial \alpha$	453.1$\mu$s	435.8$\mu$s	$\partial R / \partial \alpha$	342.3$\mu$s	407.3$\mu$s
$\partial R / \partial \theta _{xy}$	451.6$\mu$s	433.9$\mu$s	$\partial R / \partial \theta _{xy}$	410.9$\mu$s	412.9$\mu$s
$\partial R / \partial \theta _z$	457.3$\mu$s	443.6$\mu$s	$\partial R / \partial \theta _{z}$	412.7$\mu$s	416.0$\mu$s

$^a$Lenovo Thinkpad X1 (2023) 12th Gen Intel Core i7-1260P 2.10 GHz;$^b$AMD Ryzen 9 5900X 12-Core Processor 3.70 GHz

### Example optimizations

To test the performance of the analytical exact gradients, we also looked into three different optimization scenarios. The first one concerns with a relatively simple, but nevertheless important application: the development of $^{15}$N pulses that cover the full 50 ppm amide bandwidth on a 1.2 GHz spectrometer with a maximum rf-amplitude that corresponds to a rectangular 90$^{\circ }$ pulse of 50 $\mu$s. The aim was not to produce the best pulse possible, but to monitor and compare how well the analytical solutions were able to produce a good pulse and what the average duration per iteration as a measure of computation speed was. The results are summarized in Table 7 for excitation (so-called BEBOP pulses (Skinner et al. 2003, 2005; Kobzar et al. 2012)) and inversion (so-called BIBOP pulses (Ehni and Luy 2013; Kobzar et al. 2004, 2012; Nimbalkar et al. 2013)) pulse optimizations.

**Fig. 1:**
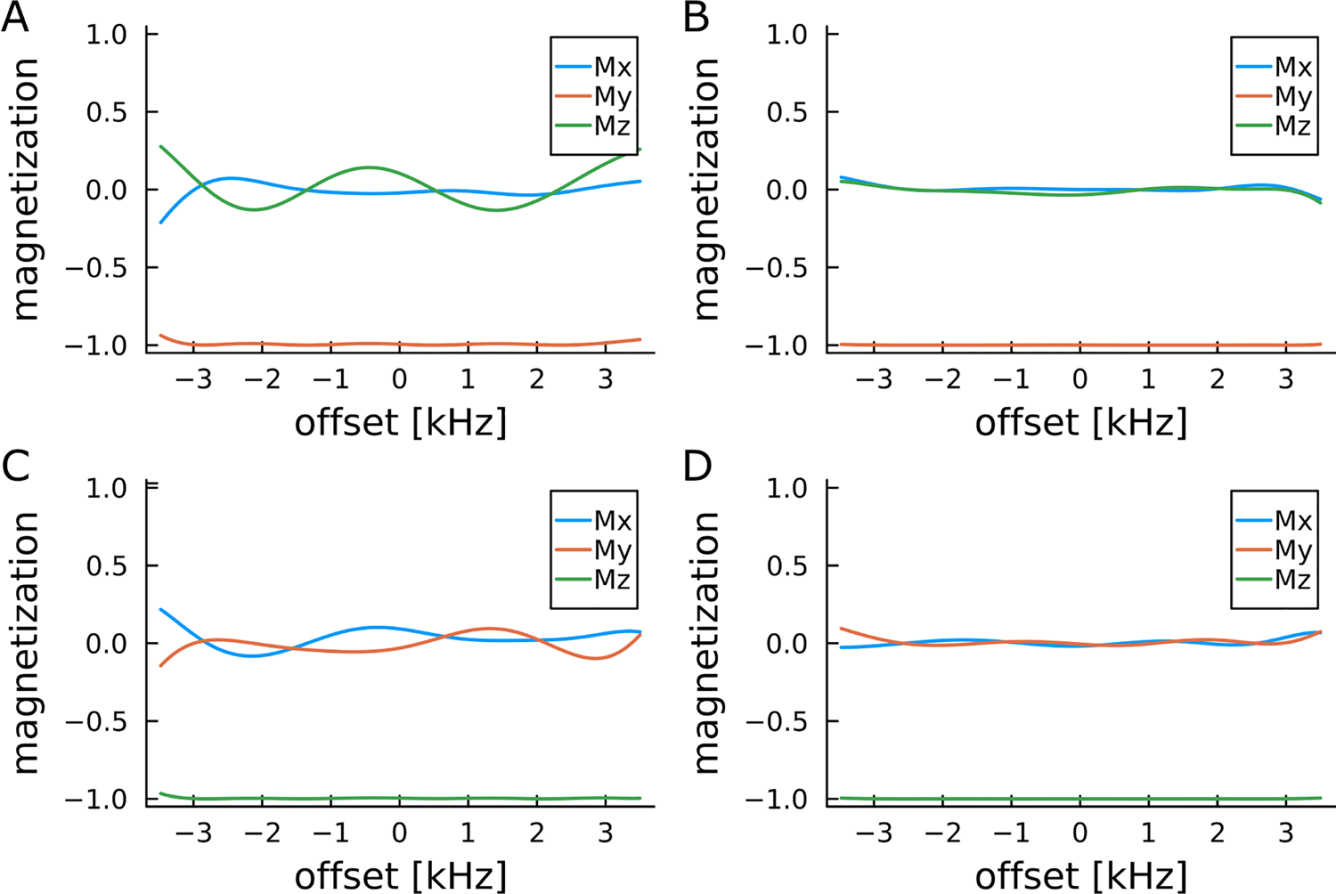
Offset profiles for excitation (**A**, **B**) and inversion (**C**, **D**) pulses optimized for amide nitrogen excitation of proteins on a 1.2 GHz spectrometer. The quality factors of the corresponding pulse shapes are written in italics in Table 7. For both types, worst (**A**, **C**) and best (**B**, **D**) pulses were simulated and differences are clearly visible

For parameters we decided to focus on amplitude-restricted pulses with a maximum rf-amplitude of 5 kHz and ±10% B$_1$-compensation. The bandwidth is 6000 kHz, which is only slightly larger than the rf-amplitude of the pulse. Pulse shapes fulfilling this condition have been optimized already as early as 2004 (Kobzar et al. 2004), although not with exactly the same parameters. For the optimization we used the Optim.jl Julia package (https://julianlsolvers.github.io/Optim.jl/stable/), with its BFGS implementation. We ran all optimizations on the very same laptop with which we compared the runtime performance in Table 6. Independent of the parameters used for optimization, the resulting pulses generally gave high quality factors larger than 0.99. Three different digitizations were used in the optimizations, where durations of the digits of 1, 10, and 50 $\mu$s led to 500, 50, and 10 piecewise constant pulse elements, respectively. It is no surprise that the latter led to fastest overall optimization times in the sub-second range, as well as the shortest computation times per iteration and also on average less iterations for convergence. Interestingly, the resulting quality factors for the short optimizations are overall in the same range as for the other optimizations. Particularly for initial screens it might therefore be of use to start a large number of fast optimizations with long $\Delta t$ elements, which then form a good basis to choose optimization parameters for a more detailed search. However, it should be noted, that there are profound differences between pulses with quality factors on the order of 0.992 and ones with quality factors of 0.999. This can nicely be seen in Fig. 1, where corresponding excitation and inversion pulses are compared in their performance. Clearly the higher quality factors correspond to significantly better performing pulse shapes.

Comparing the different controls, it is no surprise that constant amplitude optimizations that only control the pulse phase $\alpha$, resulted in shortest calculation times. This approach simply has the least amount of controls and also the smallest parameter space in the optimizations. More unexpectedly, the second fastest overall optimization times were reached by the reduced amplitude parameters $\theta _{xy}^{\textrm{red}}$ using the tanh-based restriction of equation (46). Although the additional computation of the tanh terms is computationally costly, it seems to clearly outperform the Lagrange multiplier type amplitude restriction used in the other cases. It seems therefore worth following the pseudo parameter approach when dealing with such type of restrictions.

For the more conventional approaches using either *x*, *y* or $\theta _{xy}$, $\alpha$ controls, no real difference in performance can be seen. Also the addition of *z*-controls did not really change the convergence characteristic. However, we experienced that *z*-controls lead to many optimizations that stop at a very early stage of an optimization. Apparently, the unrestricted *z*-controls lead sometimes to erratic behaviour with large frequency jumps between neighbouring digits. In principle, *z*-control should allow better convergence and a larger parameter space when rough digitizations are used in pulse optimization. The situation might change, when additional constraints like smoothness restrictions are applied to *z*-controls.

**Fig. 2:**
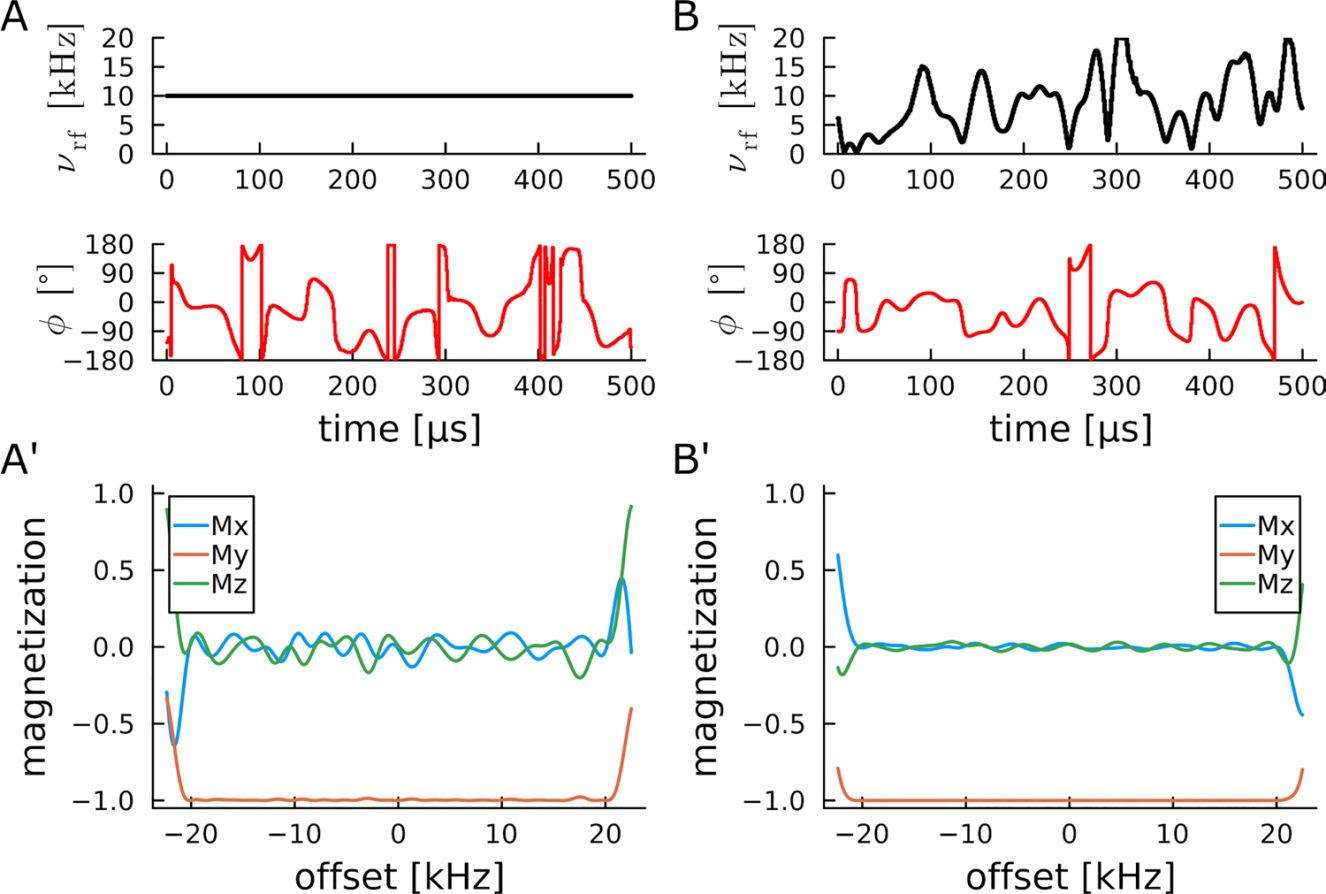
Offset profiles for two excitation pulses with identical overall rf-power (A,B) and optimization parameters given in Table 8. The best obtained BEBOP pulse shape with constant amplitude and overall quality factor 0.99715 has significantly lower performance (A’) than the power-BEBOP with quality factor 0.99982 (B’), corroborating well-known results regarding physical limits studies (Kobzar et al. 2008)

**Fig. 3:**
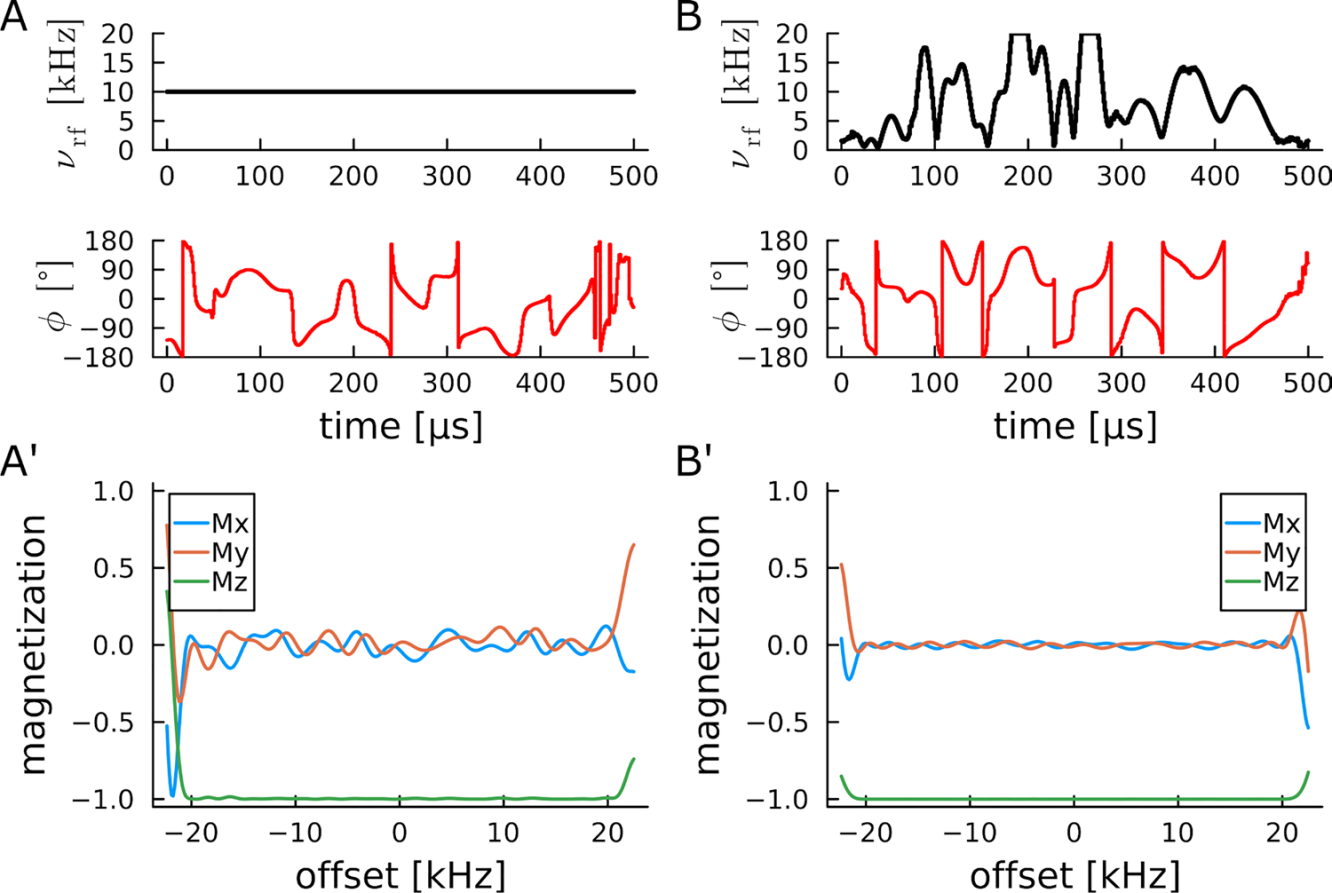
Offset profiles for two inversion pulses with identical overall rf-power (**A**, **B**) and optimization parameters given in Table 8. The best obtained BIBOP pulse shape with constant amplitude and overall quality factor 0.99744 has significantly lower performance (**A**’) than the power-BIBOP with quality factor 0.99983 (**B**’), corroborating well-known results regarding physical limits studies (Kobzar et al. 2008)

**Fig. 4:**
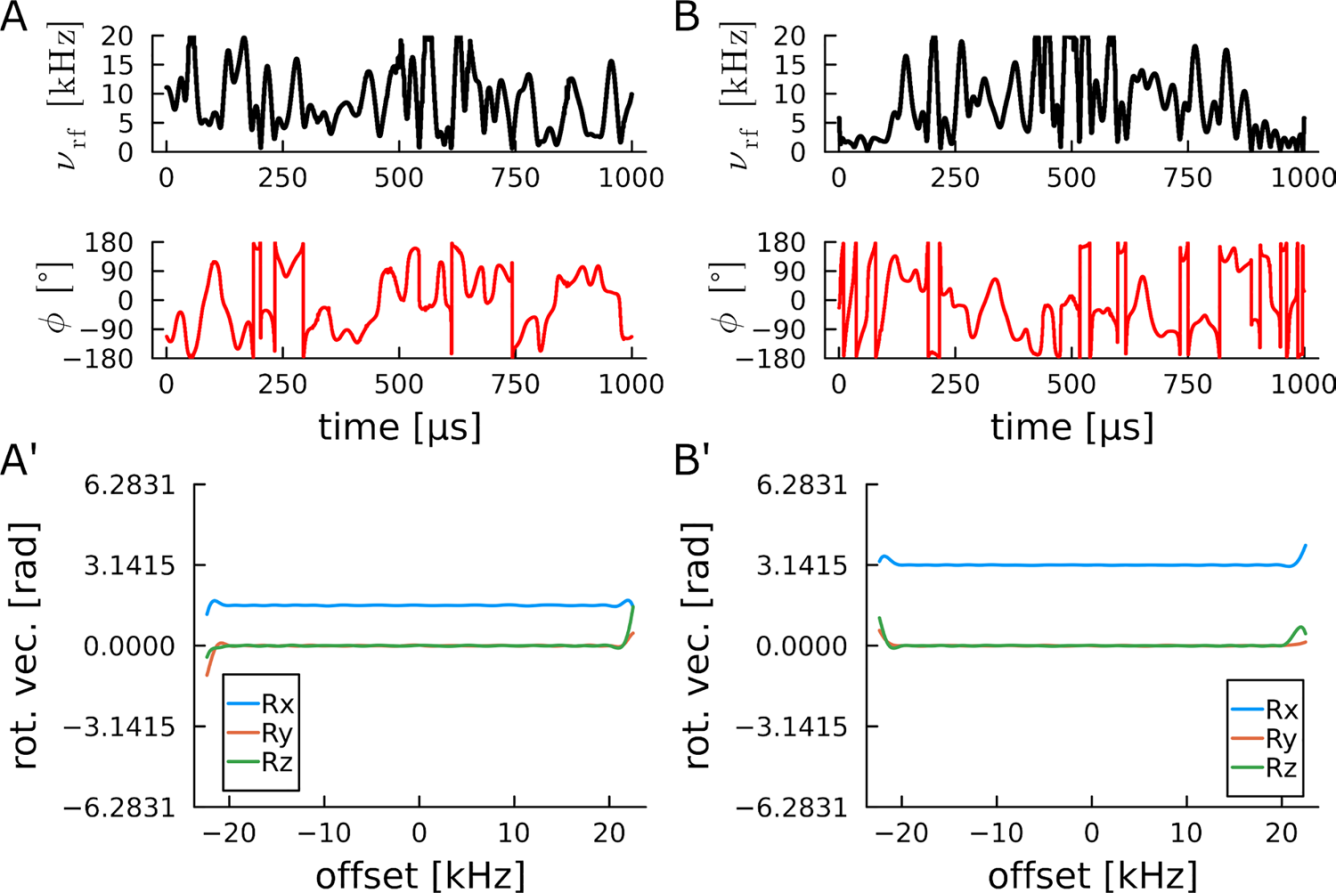
Offset profiles for a universal rotation power-BURBOP-90$^{\circ }$ (**A**) and a universal rotation power-BURBOP-180$^{\circ }$ (**B**) pulse with identical overall rf-power and optimization parameters given in Table 8. Both pulses show exceptional performance

The second studied scenario concerns pulses with a bandwidth of 40 kHz, which corresponds to a 266 ppm carbon chemical shift range on a 600 MHz spectrometer, and to 160 ppm on a 1.0 GHz spectrometer. Several pulse shapes are available covering the desired bandwidth, although usually with longer pulse shapes than needed (Kobzar et al. 2004, 2008, 2012). We optimized here a basic set of excitation, inversion and different universal rotation pulses fulfilling roughly the physical minimu durations with either constant amplitude of 10 kHz, or with a combined Lagrange multiplier type rf-amplitude (20 kHz) and rf-power ($\sqrt{\overline{P^{\textrm{max}}}}$) restrictions. Corresponding pulse shapes generally resulted in very good performance pulses, as can also be seen in Figs. 2, 3, 4 for the different pulse types. In all cases the amplitude and power-restricted pulse shapes perform better than the constant amplitude pulses with identical overall rf-power, which could be expected from the results of previous systematic studies of BEBOP/BIBOP (Kobzar et al. 2004) and power-BEBOP/power-BIBOP (Kobzar et al. 2008) pulses.

**Fig. 5:**
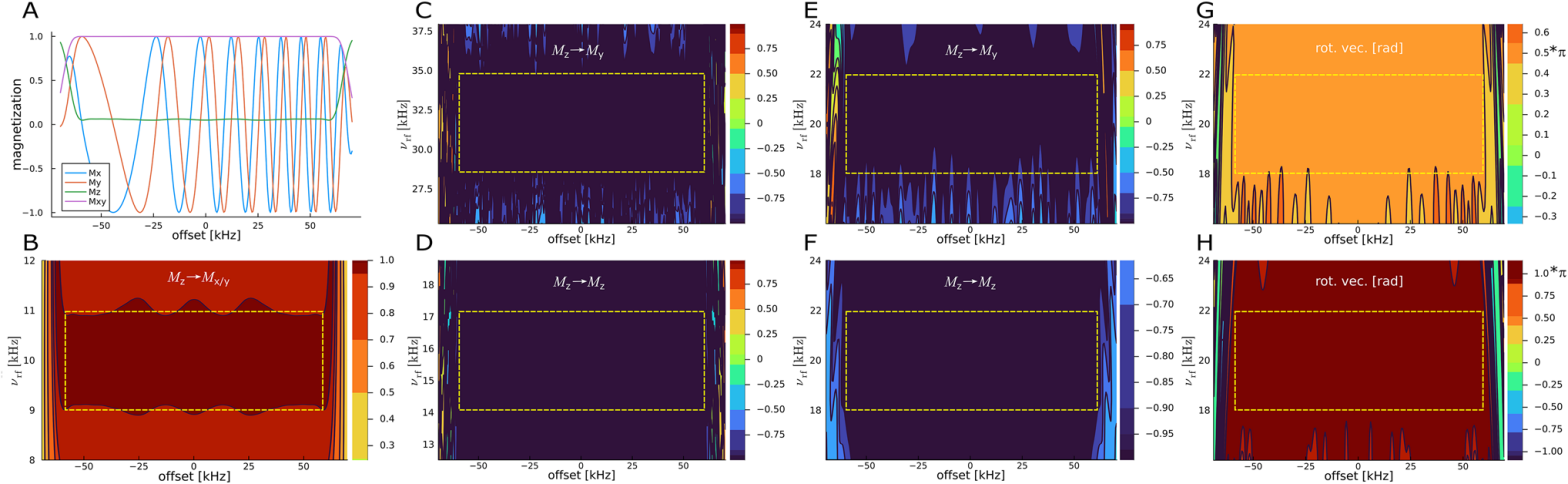
Summary of pulse shapes optimized for fluorine excitation. (**A**, **B**) extremely short saturation pulse of 120 $\mu$s covering the full bandwidth of 120 kHz. Although the pulse was optimized without B$_1$-compensation, considerable compensation is achieved in the ±10% range. (**C**, **D**) power-BEBOP excitation and power-BIBOP inversion pulses with the squareroot of the average rf-power of only 10 kHz and 1.4 ms duration. (E,F) corresponding BEBOP excitation and BIBOP inversion pulses with constant rf-amplitude of 20 kHz and durations of 350 $\mu$s and below. (G,H) Universal rotation BURBOP-90 and BURBOP-180 pulses of 600 $\mu$s and 700 $\mu$s duration equivalent to previously published pulse shapes (Lingel et al. 2020). The Offset/B$_1$ region used for most pulse optimizations is highlighted by the yellow dashed boxes

Finally, we optimized a set of pulses for $^{19}$F spectroscopy on a 600 MHz spectrometer, where previously two universal rotation pulses were optimized for screening experiments with essentially identical parameters (Lingel et al. 2020). The rational behind the optimizations is, that for other screening experiments, as well as biomolecular NMR on fluorinated samples, particularly short excitation and inversion pulses might be beneficial. The large bandwidth and considerable B$_1$-compensation for most pulses is challenging, but resulting pulse shapes lead to remarkable results, which is nicely documented in Fig. 5.

A first pulse is a saturation pulse bringing *z* magnetization efficiently into the *x*,*y* plane. As has been previously experienced (Enders et al. 2014; Luy et al. 2009; Woordes et al. 2025), the wide offset range can be covered with a very short pulse shape. It should also be noted that the overall optimization time of this difficult pulse on a single processor core required only 1.6 seconds with a close to perfect performance. Although no B$_1$-compensation was applied, the pulse will be very well applicable, as can be seen in Fig. 5B. Surprisingly good are also the excitation and inversion pulses with their overall rf-power restricted to the equivalent to a 10 kHz constant amplitude pulse. The restriction in this case has been applied via the tanh-type pseudo controls with inherent power-based scaling. Both pulse shapes with a high number of offset checks, 700 piecewise constant pulse digits, and detailed B$_1$-compensation were the most complex optimizations attempted here. Correspondingly, optimization times comprise half a day to a day on a single core. The bandwidth-over-B$_1$-ratio in this case is 12, which is much larger than for most previously optimized excitation and inversion pulses, and demonstrates to some extent the increase in complexity and optimization duration with more demanding pulse shapes. Both optimizations did not fully converge, but stopped after the maximum number of iterations specified (5000 iterations). It can be expected that optimizations that run until convergence is reached will lead to better pulse shapes, but most likely with only slight improvements. Finally, constant amplitude pulse shapes with a bandwidth-over-B$_1$-ratio of 6 were optimized for excitation, inversion, and universal rotation 90$^{\circ }$ and 180$^{\circ }$ pulses. All of the pulses are rather short for their performance and should be readily suited for corresponding $^{19}$F-based applications.

## Conclusion

Cost functions and corresponding exact gradients for point-to-point and universal rotation type single spin optimizations have been derived analytically. Their implementation into the self-written program PulseOptimizer (will be published somewhere else) resulted into improvements in computation times compared to other analytical gradient calculations like the augmented matrix exponentiation approach by roughly two orders of magnitude. Correspondingly, very different types of conventional pulse shapes could be optimized in a matter of seconds to few hours using a single processor core on a laptop. With this increase in optimization speed an important step towards the optimization of pulse shapes with very large bandwidth-over-B$_1$-ratios is done. However, already some of the example pulses have shown that further technical improvements in optimization software will be necessary to tackle pulses with bandwidths that are 100 times larger than the corresponding B$_1$ field strenghts. As such, massive parallelization and eventually efficient implementation in GPU architectures either on a quality factor level and/or on an optimizer level seem to be appropriate additional future steps.

**Table 7:** Summary of individual optimizations performed using the different formulae from Tables 1-3. In all cases, pulses of 500 $\mu$s duration, maximum rf-amplitude of 5 kHz with ±10% B$_1$-compensation (3 $B_1$ points), and a bandwidth of 6000 kHz (11 offset points, covering 50 ppm in $^{15}$N at a 1.2 GHz spectrometer) were optimized using a BFGS optimization algorithm on a single core of a laptop

Controls	$\Delta$t	Iterations	Optimization	Time per	Quality
	[$\mu$s]	#	time [s]	iter. [$\mu$s]	factor
Excitation					
$\theta _x$,$\theta _y$	1/10/50	4944/424/148	665,4/7,4/0,598	134,5/17,4/4,0	0,9987/0,9960/0,9973
$\theta _x$,$\theta _y$,$\theta _z$	1/10/50	1294/152/98	306,2/7,2/0,277	236,6/47,3/2,8	0,9990/0,9938/0,9955
$\theta _{xy}$,$\alpha$	1/10/50	777/134/119	383,9/7,6/0,608	494,0/56,7/5,1	0,9962/0,9949/*0,9923*
$\theta _{xy}$,$\alpha$,$\theta _z$	1/10/50	1083/198/65	991,3/12,8/0,428	915,3/64,6/6,5	0,9978/0,9981/0,9968
$\theta _{xy}^{\textrm{red}}$,$\alpha$	1/10/50	147/146/105	17,7/2,8/0,407	120,4/19,1/3,8	0,9966/0,9991/0,9985
$\theta _{xy}^{\textrm{red}}$,$\alpha$,$\theta _z$	1/10/50	765/247/85	82,7/5,5/0,421	108,1/22,2/4,9	0,9989/0,9989/0,9968
$\alpha$	1/10/50	847/1018/82	53,7/8,1/0,126	63,4/7,9/1,5	0,9981/*0,9995*/0,9963
Inversion					
$\theta _x$,$\theta _y$	1/10/50	4805/231/87	727.4/5.4/0.277	151.3/23.3/3.1	0.9978/0.9947/0.9926
$\theta _x$,$\theta _y$,$\theta _z$	1/10/50	1427/185/70	316.7/6.8/0.418	221.9/36.7/5.9	0.9972/0.9971/0.9946
$\theta _{xy}$,$\alpha$	1/10/50	809/1192/78	488.6/21/0.318	603.9/17.6/4.0	*0.9926*/0.9996/0.9972
$\theta _{xy}$,$\alpha$,$\theta _z$	1/10/50	819/173/132	632.9/11.3/0.74	772.7/65.3/5.6	0.9950/0.9961/0.9987
$\theta _{xy}^{\textrm{red}}$,$\alpha$	1/10/50	331/302/95	46.2/3.5/0.376	139.5/11.5/3.9	0.9972/0.9995/0.9973
$\theta _{xy}^{\textrm{red}}$,$\alpha$,$\theta _z$	1/10/50	260/121/54	40.8/2.5/0.228	156.9/20.6/4.2	0.9965/0.9995/0.9926
$\alpha$	1/10/50	573/753/88	39.2/4.4/0.215	68.4/5.8/2.4	*0.9997*/0.9981/0.9932

**Table 8:** Different pulse types optimized for a typical $^{13}$C scenario, where 250 ppm on a 600 MHz spectrometer or 160 ppm on a 1.0 GHz spectrometer have to be covered. Pulses were optimized using an L-BFGS optimization algorithm on a single core of a laptop

Pulse type	Controls	Offset	BW	B$_1$	$\pm \vartheta$	$\Delta$t	$t_p$	$\sqrt{\overline{P}^{\textrm{max}}}$/	iter.	opt.	Quality
		#	[kHz]	#	[%]	[$\mu$s]	[$\mu$s]	$\theta _{xy}^{\textrm{max}}$[kHz]	#	time [s]	factor
excitation	$\theta _x$,$\theta _y$	31	40	3	5	1	500	10/20	3956	1664.0	*0.99982*
excitation	$\theta _x$,$\theta _y$,$\theta _z$	31	40	3	5	10	500	10/20	90	6.4	0.99914
excitation	$\alpha$	31	40	3	5	1	500	-/10	1248	293.0	*0.99715*
inversion	$\theta _x$,$\theta _y$	31	40	3	5	1	500	10/20	1966	690.0	*0.99983*
inversion	$\theta _x$,$\theta _y$,$\theta _z$	31	40	3	5	10	500	10/20	104	7.5	0.99839
inversion	$\alpha$	31	40	3	5	1	500	-/10	1473	344.0	*0.99744*
UR-90$^{\circ }$	$\theta _x$,$\theta _y$	61	40	3	5	1	1000	10/20	5000$^*$	3170.0	*0.99996*
UR-180$^{\circ }$	$\theta _x$,$\theta _y$	61	40	3	5	1	1000	10/20	2854	3353.6	*0.99998*

$^*$optimization stopped after reaching maximum number of iterations

**Table 9:** Different pulse types optimized for a typical $^{19}$F scenario, where 200 ppm need to be covered on a 600 MHz spectrometer. Pulses were optimized using an L-BFGS optimization algorithm on a single core of a laptop

Pulse type	Controls	Offset	BW	B$_1$	$\pm \vartheta$	$\Delta$t	$t_p$	$\sqrt{\overline{P}^{\textrm{max}}}$/	iter.	Opt.	Quality
		#	[kHz]	#	[%]	[$\mu$s]	[$\mu$s]	$\theta _{xy}^{\textrm{max}}$[kHz]	#	time [s]	Factor
saturation	$\alpha$	31	120	1	-	2	120	-/10	244	1.6	*0.99999*
excitation	$\theta _{xy}^{\textrm{red}}$,$\alpha$	301	120	5	10	2	1400	10/-	5000$^*$	94570	*0.99627*
excitation	$\alpha$	121	120	5	10	1	350	-/20	894	904.4	*0.99419*
inversion	$\theta _{xy}^{\textrm{red}}$,$\alpha$	301	120	5	10	2	1400	10/-	5000$^*$	42864	*0.99989*
inversion	$\alpha$	121	120	5	10	1	300	-/20	567	757.1	*0.99885*
UR-90$^{\circ }$	$\alpha$	121	120	5	10	1	600	-/20	2587	2833.2	*0.99915*
UR-180$^{\circ }$	$\alpha$	121	120	5	10	1	700	-/20	10000$^*$	15411.0	*0.99951*

$^*$optimization stopped after reaching maximum number of iterations

## Supplementary Information

Below is the link to the electronic supplementary material.

Supplementary file 1 (zip 804 KB)

## Data Availability

No datasets were generated or analysed during the current study.

## References

[CR1] Aizu K (1963) Parameter differentiation of quantum-mechanical linear operators. J Math Phys 4(6):762–775. 10.1063/1.1724318

[CR2] Bonnard B, Glaser SJ, Sugny D (2012) A review of geometric optimal control for quantum systems in nuclear magnetic resonance. Adv Math Phys 2012(1):857493. 10.1155/2012/857493

[CR3] Buchanan CJ, Bhole G, Karunanithy G, Casablancas-Antràs V, Poh AWJ, Davis BG et al (2025) Seedless: on-the-fly pulse calculation for NMR experiments. Nat Commun 16(1):7276. 10.1038/s41467-025-61663-8 40775231 10.1038/s41467-025-61663-8 PMC12332029

[CR4] Cayley A IV (1997) A sixth memoir upon quantics. Philos Trans R Soc Lond 149:61–90. 10.1098/rstl.1859.0004

[CR5] Conolly S, Nishimura D, Macovski A (1986) Optimal control solutions to the magnetic resonance selective excitation problem. IEEE Trans Med Imaging 5(2):106–115. 10.1109/TMI.1986.4307754 18243994 10.1109/TMI.1986.4307754

[CR6] de Fouquieres P, Schirmer SG, Glaser SJ, Kuprov I (2011) Second order gradient ascent pulse engineering. J Magn Reson 212(2):412–417. 10.1016/j.jmr.2011.07.023 21885306 10.1016/j.jmr.2011.07.023

[CR7] Ehni S, Koos MRM, Reinsperger T, Haller JD, Goodwin DL, Luy B (2022) Concurrent J-evolving refocusing pulses. J Magn Reson 336:107152. 10.1016/j.jmr.2022.107152 35189510 10.1016/j.jmr.2022.107152

[CR8] Ehni S, Luy B (2012) A systematic approach for optimizing the robustness of pulse sequence elements with respect to couplings, offsets, and -field inhomogeneities (COB). Magn Reson Chem. 50(S1):S63–S72. 10.1002/mrc.3846 23280662 10.1002/mrc.3846

[CR9] Ehni S, Luy B (2013) BEBEtr and BUBI: j-compensated concurrent shaped pulses for 1H–13C experiments. J Magn Reson 232:7–17. 10.1016/j.jmr.2013.04.007 23673080 10.1016/j.jmr.2013.04.007

[CR10] Ehni S, Luy B (2014) Robust INEPT and refocused INEPT transfer with compensation of a wide range of couplings, offsets, and B 1 -field inhomogeneities (COB3). J Magn Reson 247:111–117. 10.1016/j.jmr.2014.07.010 25245402 10.1016/j.jmr.2014.07.010

[CR11] Emsley L, Bodenhausen G (1990) Gaussian pulse cascades: new analytical functions for rectangular selective inversion and in-phase excitation in NMR. Chem Phys Lett 165(6):469–476. 10.1016/0009-2614(90)87025-M

[CR12] Emsley L, Bodenhausen G (1992) Optimization of shaped selective pulses for NMR using a quaternion description of their overall propagators. J Magn Reson 97(1):135–148. 10.1016/0022-2364(92)90242-Y

[CR13] Enders M, Görling B, Braun AB, Seltenreich JE, Reichenbach LF, Rissanen K et al (2014) Cytotoxicity and NMR studies of platinum complexes with cyclooctadiene ligands. Organometallics 33(15):4027–4034. 10.1021/om500540x

[CR14] Ewing B, Glaser SJ, Drobny GP (1990) Development and optimization of shaped NMR pulses for the study of coupled spin systems. Chem Phys 147(1):121–129. 10.1016/0301-0104(90)85028-U

[CR15] Floether FF, Fouquieres PD, Schirmer SG (2012) Robust quantum gates for open systems via optimal control: markovian versus non-markovian dynamics. New J Phys 14(7):073023. 10.1088/1367-2630/14/7/073023

[CR16] Foroozandeh M, Singh P (2021) Optimal control of spins by analytical lie algebraic derivatives. Automatica 129:109611. 10.1016/j.automatica.2021.109611

[CR17] Garwood M, Ke Y (1991) Symmetric pulses to induce arbitrary flip angles with compensation for Rf inhomogeneity and resonance offsets. Journal of Magnetic Resonance (1969) 94(3):511–525. 10.1016/0022-2364(91)90137-I

[CR18] Gershenzon NI, Kobzar K, Luy B, Glaser SJ, Skinner TE (2007) Optimal control design of excitation pulses that accommodate relaxation. J Magn Reson 188(2):330–336. 10.1016/j.jmr.2007.08.007 17804269 10.1016/j.jmr.2007.08.007

[CR19] Gershenzon NI, Skinner TE, Brutscher B, Khaneja N, Nimbalkar M, Luy B et al (2008) Linear phase slope in pulse design: application to coherence transfer. J Magn Reson 192(2):235–243. 10.1016/j.jmr.2008.02.021 18394937 10.1016/j.jmr.2008.02.021

[CR20] Goodwin DL, Kuprov I (2015) Auxiliary matrix formalism for interaction representation transformations, optimal control, and spin relaxation theories. J Chem Phys 143(8):084113. 10.1063/1.4928978 26328824 10.1063/1.4928978

[CR21] Goodwin DL, Kuprov I (2016) Modified Newton-Raphson GRAPE methods for optimal control of spin systems. J Chem Phys 144(20):204107. 10.1063/1.4949534 27250279 10.1063/1.4949534

[CR22] Goodwin DL (2017) Advanced optimal control methods for spin systems. PhD thesis

[CR23] Goodwin DL, Vinding MS (2023) Accelerated Newton-Raphson GRAPE methods for optimal control. Phys Rev Res 5(1):L012042. 10.1103/PhysRevResearch.5.L012042

[CR24] Haller JD, Goodwin DL, Luy B (2022) SORDOR pulses: expansion of the Böhlen-Bodenhausen scheme for low-power broadband magnetic resonance. Magn Reson 3(1):53–63. 10.5194/mr-3-53-2022 10.5194/mr-3-53-2022 PMC10539771 37905174

[CR25] Hamilton WR (1843) On quaternions; or on a new system of imaginaries in algebra. Letter to John T. Graves

[CR26] He M, Faderl D, MacKinnon N, Cheng YT, Buyens D, Jouda M et al (2024) A digital twin for parallel liquid-state nuclear magnetic resonance spectroscopy. Commun Eng 3(1):1–13. 10.1038/s44172-024-00233-0

[CR27] Hogben HJ, Krzystyniak M, Charnock GTP, Hore PJ, Kuprov I (2011) Spinach - A Software Library for Simulation of Spin Dynamics in Large Spin Systems. J Magn Reson 208(2):179–194. 10.1016/j.jmr.2010.11.008 21169043 10.1016/j.jmr.2010.11.008

[CR28] Joseph D, Griesinger C (2023) Optimal Control Pulses for the 1.2-GHz (28.2-T) NMR Spectrometers. Sci Adv 9(45):eadj1133. 10.1126/sciadv.adj1133 37948513 10.1126/sciadv.adj1133 PMC10637738

[CR29] Kadkhodaie M, Rivas O, Tan M, Mohebbi A, Shaka AJ (1991) Broadband Homonuclear Cross Polarization Using Flip-Flop Spectroscopy. Journal of Magnetic Resonance. 91(2):437–443. 10.1016/0022-2364(91)90210-K

[CR30] Khaneja N, Reiss T, Kehlet C, Schulte-Herbrüggen T, Glaser SJ (2005) Optimal Control of Coupled Spin Dynamics: Design of NMR Pulse Sequences by Gradient Ascent Algorithms. J Magn Reson 172(2):296–305. 10.1016/j.jmr.2004.11.004 15649756 10.1016/j.jmr.2004.11.004

[CR31] Kobzar K, Ehni S, Skinner TE, Glaser SJ, Luy B (2012) Exploring the Limits of Broadband 90 and 180 Universal Rotation Pulses. J Magn Reson 225:142–160. 10.1016/j.jmr.2012.09.013 23142001 10.1016/j.jmr.2012.09.013

[CR32] Kobzar K, Luy B, Khaneja N, Glaser SJ (2005) Pattern Pulses: Design of Arbitrary Excitation Profiles as a Function of Pulse Amplitude and Offset. J Magn Reson 173(2):229–235. 10.1016/j.jmr.2004.12.005 15780915 10.1016/j.jmr.2004.12.005

[CR33] Kobzar K, Skinner TE, Khaneja N, Glaser SJ, Luy B (2004) Exploring the Limits of Broadband Excitation and Inversion Pulses. J Magn Reson 170(2):236–243. 10.1016/j.jmr.2004.06.017 15388086 10.1016/j.jmr.2004.06.017

[CR34] Kobzar K, Skinner TE, Khaneja N, Glaser SJ, Luy B (2008) Exploring the Limits of Broadband Excitation and Inversion: II. Rf-power Optimized Pulses J Magn Reson 194(1):58–66. 10.1016/j.jmr.2008.05.023 18586540 10.1016/j.jmr.2008.05.023

[CR35] Koos MRM, Feyrer H, Luy B (2015) Broadband excitation pulses with variable RF amplitude-dependent flip angle (RADFA). Magn Reson Chem 53(11):886–893. 10.1002/mrc.4297 26259565 10.1002/mrc.4297

[CR36] Koos MRM, Feyrer H, Luy B (2017) Broadband RF-amplitude-dependent flip angle pulses with linear phase slope. Magn Reson Chem 55(9):797–803. 10.1002/mrc.4593 28321918 10.1002/mrc.4593

[CR37] Kupce E, Freeman R (1993) Polychromatic selective pulses. J Magn Reson Ser A 102(1):122–126. 10.1006/jmra.1993.1079

[CR38] Kupce E, Freeman R (1994) Wideband excitation with polychromatic pulses. J Magn Reson Ser A 108(2):268–273. 10.1006/jmra.1994.1123

[CR39] Kuprov I (2023) Optimal Control of Spin Systems. In: KUPROV ILYA, editor. Spin: From Basic Symmetries to Quantum Optimal Control. Cham: Springer International Publishing. p 313–349

[CR40] Lapert M, Zhang Y, Janich MA, Glaser SJ, Sugny D (2012) Exploring the physical limits of saturation contrast in magnetic resonance imaging. Sci Rep 2(1):589. 10.1038/srep00589

[CR41] Levante TO, Bremi T, Ernst RR (1996) Pulse-sequence optimization with analytical derivatives. Application to deuterium decoupling in oriented phases. J Magn Reson Ser A 121(2):167–177. 10.1006/jmra.1996.0157

[CR42] Levitt MH (1986) Composite pulses. Prog Nucl Magn Reson Spectrosc 18(2):61–122. 10.1016/0079-6565(86)80005-X

[CR43] Levitt MH, Freeman R (1979) NMR population inversion using a composite pulse. J Magn Reson 33(2):473–476. 10.1016/0022-2364(79)90265-8 10.1016/j.jmr.2011.08.016 21903438

[CR44] Levitt MH, Freeman R, Frenkiel T (1982) Broadband heteronuclear decoupling. J Magn Reson 47(2):328–330. 10.1016/0022-2364(82)90124-X

[CR45] Lingel A, Vulpetti A, Reinsperger T, Proudfoot A, Denay R, Frommlet A et al (2020) Comprehensive and high-throughput exploration of chemical space using broadband ^19^F NMR-based screening. Angew Chem Int Ed 59(35):14809–14817. 10.1002/anie.202002463 10.1002/anie.202002463 32363632

[CR46] Lurie DJ (1986) Numerical design of composite radiofrequency pulses. J Magn Reson 70(1):11–20. 10.1016/0022-2364(86)90359-8

[CR47] Luy B, Kobzar K, Glaser SJ, Khaneja N (2009) Ultrabroadband NMR Spectroscopy Using Xy-BEBOP Saturation Pulses. In: 50th Experimental NMR Conference Pacific Grove, CA

[CR48] Luy B, Kobzar K, Skinner TE, Khaneja N, Glaser SJ (2005) Construction of universal rotations from point-to-point transformations. J Magn Reson 176(2):179–186. 10.1016/j.jmr.2005.06.002 16009584 10.1016/j.jmr.2005.06.002

[CR49] Mao J, Mareci TH, Scott KN, Andrew ER (1986) Selective inversion radiofrequency pulses by optimal control. J Magn Reson 70(2):310–318. 10.1016/0022-2364(86)90016-8

[CR50] Martikyan V, Beluffi C, Glaser SJ, Delsuc MA, Sugny D (2021) Application of optimal control theory to Fourier transform ion cyclotron resonance. Molecules 26(10):2860. 10.3390/molecules26102860 34065881 10.3390/molecules26102860 PMC8151339

[CR51] Neves JL, Heitmann B, Khaneja N, Glaser SJ (2009) Heteronuclear decoupling by optimal tracking. J Magn Reson 201(1):7–17. 10.1016/j.jmr.2009.07.024 19695913 10.1016/j.jmr.2009.07.024

[CR52] Nimbalkar M, Luy B, Skinner TE, Neves JL, Gershenzon NI, Kobzar K et al (2013) The fantastic four: a plug ‘n’ play set of optimal control pulses for enhancing NMR spectroscopy. J Magn Reson 228:16–31. 10.1016/j.jmr.2012.12.007 23333616 10.1016/j.jmr.2012.12.007

[CR53] Odedra S, Wimperis S (2012) Use of composite refocusing pulses to form spin echoes. J Magn Reson 214:68–75. 10.1016/j.jmr.2011.10.006 22070969 10.1016/j.jmr.2011.10.006

[CR54] Rodrigues O (1840) Des Lois Géométriques Qui Régissent Les Déplacements d’un Système Solide Dans l’espace, et de La Variation Des Coordonnées Provenant de Ces Déplacements Considérés Indépendamment Des Causes Qui Peuvent Les Produire. Journal de mathématiques pures et appliquées. p 380–440

[CR55] Rosenfeld D, Zur Y (1996) Design of adiabatic selective pulses using optimal control theory. Magn Reson Med 36(3):401–409. 10.1002/mrm.1910360311 8875410 10.1002/mrm.1910360311

[CR56] Schilling F, Warner LR, Gershenzon NI, Skinner TE, Sattler M, Glaser SJ (2014) Next-generation heteronuclear decoupling for high-field biomolecular NMR spectroscopy. Angew Chem Int Ed 53(17):4475–4479. 10.1002/anie.201400178 10.1002/anie.201400178 24623579

[CR57] Shaka AJ, Keeler J, Freeman R (1983) Evaluation of a new broadband decoupling sequence: WALTZ-16. J Magn Reson 53(2):313–340. 10.1016/0022-2364(83)90035-5

[CR58] Shaka AJ, Lee CJ, Pines A (1988) Iterative schemes for bilinear operators; application to spin decoupling. J Magn Reson 77(2):274–293. 10.1016/0022-2364(88)90178-3

[CR59] Skinner TE, Gershenzon NI, Nimbalkar M, Bermel W, Luy B, Glaser SJ (2012) New strategies for designing robust universal rotation pulses: application to broadband refocusing at low power. J Magn Reson 216:78–87. 10.1016/j.jmr.2012.01.005 22325853 10.1016/j.jmr.2012.01.005

[CR60] Skinner TE, Kobzar K, Luy B, Bendall MR, Bermel W, Khaneja N et al (2006) Optimal control design of constant amplitude phase-modulated pulses: application to calibration-free broadband excitation. J Magn Reson 179(2):241–249. 10.1016/j.jmr.2005.12.010 16413802 10.1016/j.jmr.2005.12.010

[CR61] Skinner TE, Reiss TO, Luy B, Khaneja N, Glaser SJ (2003) Application of Optimal Control Theory to the Design of Broadband Excitation Pulses for High-Resolution NMR. J Magn Reson 163(1):8–15. 10.1016/S1090-7807(03)00153-8 12852902 10.1016/s1090-7807(03)00153-8

[CR62] Skinner TE, Reiss TO, Luy B, Khaneja N, Glaser SJ (2004) Reducing the Duration of Broadband Excitation Pulses Using Optimal Control with Limited RF Amplitude. J Magn Reson 167(1):68–74. 10.1016/j.jmr.2003.12.001 14987600 10.1016/j.jmr.2003.12.001

[CR63] Skinner TE, Reiss TO, Luy B, Khaneja N, Glaser SJ (2005) Tailoring the Optimal Control Cost Function to a Desired Output: Application to Minimizing Phase Errors in Short Broadband Excitation Pulses. J Magn Reson 172(1):17–23. 10.1016/j.jmr.2004.09.011 15589403 10.1016/j.jmr.2004.09.011

[CR64] Slad S, Bermel W, Kümmerle R, Mathieu D, Luy B (2022) Band-Selective Universal 90 and 180 Rotation Pulses Covering the Aliphatic Carbon Chemical Shift Range for Triple Resonance Experiments on 1.2 GHz Spectrometers. J Biomol NMR 76(5–6):185–195. 10.1007/s10858-022-00404-1 36418752 10.1007/s10858-022-00404-1 PMC9712393

[CR65] Smith MA, Hu H, Shaka AJ (2001) Improved Broadband Inversion Performance for NMR in Liquids. J Magn Reson 151(2):269–283. 10.1006/jmre.2001.2364

[CR66] Spindler PE, Glaser SJ, Skinner TE, Prisner TF (2013) Broadband Inversion PELDOR Spectroscopy with Partially Adiabatic Shaped Pulses. Angew Chem Int Ed 52(12):3425–3429. 10.1002/anie.201207777 10.1002/anie.201207777 23424088

[CR67] Spindler PE, Schöps P, Kallies W, Glaser SJ, Prisner TF (2017) Perspectives of Shaped Pulses for EPR Spectroscopy. J Magn Reson 280:30–45. 10.1016/j.jmr.2017.02.023 28579101 10.1016/j.jmr.2017.02.023

[CR68] Spindler PE, Zhang Y, Endeward B, Gershernzon N, Skinner TE, Glaser SJ et al (2012) Shaped Optimal Control Pulses for Increased Excitation Bandwidth in EPR. J Magn Reson 218:49–58. 10.1016/j.jmr.2012.02.013 22578555 10.1016/j.jmr.2012.02.013

[CR69] Stockmann JP, Wald LL (2018) In Vivo B0 Field Shimming Methods for MRI at 7 T. Neuroimage 168:71–87. 10.1016/j.neuroimage.2017.06.013 28602943 10.1016/j.neuroimage.2017.06.013 PMC5760477

[CR70] Tannús A, Garwood M (1997) Adiabatic pulses. NMR Biomed 10(8):423–434. https://doi.org/10.1002/(SICI)1099-1492(199712)10:8<423::AID-NBM488>3.0.CO;2-X 9542739 10.1002/(sici)1099-1492(199712)10:8<423::aid-nbm488>3.0.co;2-x

[CR71] Tycko R, Cho HM, Schneider E, Pines A (1985) Composite pulses without phase distortion. J Magn Reson 61(1):90–101. 10.1016/0022-2364(85)90270-7

[CR72] Van Loan C (1978) Computing integrals involving the matrix exponential. IEEE Trans Automat Control 23(3):395–404. 10.1109/TAC.1978.1101743

[CR73] Vinding MS, Guérin B, Vosegaard T, Nielsen NC (2017) Local SAR, global SAR, and power-constrained large-flip-angle pulses with optimal control and virtual observation points. Magn Reson Med 77(1):374–384. 10.1002/mrm.26086 26715084 10.1002/mrm.26086 PMC4929033

[CR74] Vinding MS, Goodwin DL, Kuprov I, Lund TE (2021) Optimal control gradient precision trade-offs: application to fast generation of DeepControl libraries for MRI. J Magn Reson 333:107094. 10.1016/j.jmr.2021.107094 34794089 10.1016/j.jmr.2021.107094

[CR75] Woordes YT, Kobzar K, Ehni S, Görling B, Schilling F, Pianowski ZL, et al (2025) Ultrabroadband 1D and 2D NMR Spectroscopy. Angew Chem Int Ed. p. accepted. Preprint: 10.26434/chemrxiv-2025-b2zhn 10.1002/anie.202515467 PMC12790362 41307299

[CR76] Zax DB, Goelman G, Vega S (1988) Amplitude-modulated composite pulses. J Magn Reson 80(2):375–382. 10.1016/0022-2364(88)90312-5

